# A large scale screening study with a SMR-based BCI: Categorization of BCI users and differences in their SMR activity

**DOI:** 10.1371/journal.pone.0207351

**Published:** 2019-01-25

**Authors:** Claudia Sannelli, Carmen Vidaurre, Klaus-Robert Müller, Benjamin Blankertz

**Affiliations:** 1 Department of Neurotechnology, Technische Universität Berlin, Berlin, Germany; 2 Department of Machine Learning, Technische Universität Berlin, Berlin, Germany; 3 Department of Mathematics, Public University of Navarre, Pamplona, Spain; 4 Department of Brain and Cognitive Engineering, Korea University, Seoul, South Korea; 5 Max Planck Institute for Informatics, Saarbrücken, Germany; Drexel University, UNITED STATES

## Abstract

Brain-Computer Interfaces (BCIs) are inefficient for a non-negligible part of the population, estimated around 25%. To understand this phenomenon in Sensorimotor Rhythm (SMR) based BCIs, data from a large-scale screening study conducted on 80 novice participants with the Berlin BCI system and its standard machine-learning approach were investigated. Each participant performed one BCI session with resting state Encephalography, Motor Observation, Motor Execution and Motor Imagery recordings and 128 electrodes. A significant portion of the participants (40%) could not achieve BCI control (feedback performance > 70%). Based on the performance of the calibration and feedback runs, BCI users were stratified in three groups. Analyses directed to detect and elucidate the differences in the SMR activity of these groups were performed. Statistics on reactive frequencies, task prevalence and classification results are reported. Based on their SMR activity, also a systematic list of potential reasons leading to performance drops and thus hints for possible improvements of BCI experimental design are given. The categorization of BCI users has several advantages, allowing researchers 1) to select subjects for further analyses as well as for testing new BCI paradigms or algorithms, 2) to adopt a better subject-dependent training strategy and 3) easier comparisons between different studies.

## Introduction

A Brain-Computer Interface (BCI), proposed for the first time by Vidal, [1], establishes an alternative pathway between a person and a device translating the brain activity in a control command for the device, i.e. decoding the human intention and bypassing the normal motor pathways [2–8]. One common type of BCI is based on the voluntary modulation of Sensorimotor Rhythm (SMR). It exploits the Event-Related Desynchronization/Event-Related Synchronization (ERD/ERS) [9] observed in the encephalographic data (EEG) during Motor Imagery (MI) of different limbs. Within SMR-based systems, the Berlin Brain-Computer Interface (BBCI) approach could boost classification performance by introducing a calibration recording (i.e. MI trials without feedback) to train filters extracted using the Common Spatial Patterns technique (CSP) in the very same BCI session [10–13]. Still a non-negligible number of users exhibiting poor performance was reported in each study. The percentage of such BCI users is in general established to be 10-50% [14]. In particular, it has been reported that about 20% of users is not able to gain BCI control, while another 30% obtains just poor control [15–17]. Usually, SMR-based BCI systems need longer user training or a co-adaptive approach [18–22] to achieve a similar level of control than ERP or SSVEP based BCIs [23].

We name this phenomenon “BCI inefficiency” [17] (this was earlier called BCI illiteracy, see Conclusions section) referring to the inability of BCI systems to successfully deal with the brain signals of all BCI users. Several studies investigated the causes influencing the learning process of the BCI control in a neuro-feedback paradigm and what can predict it. Early studies focused on the psychological user state to explain performance variations, other studies aimed to assess individual characteristics through questionnaires. In [24] it was shown that memory span, personality factors and “dealing with stressful situations” could predict BCI performance. [25] showed that the initial performance could predict future performance in a BCI based on Slow Cortical Potentials within a sample of five severely paralyzed patients. These results were replicated by [26]. [27] reported a significant correlation between the SMR-BCI feedback performance and “locus of control by dealing with technology”. Correlation between mood and motivation with SMR-BCI performance was evaluated in a sample of 16 healthy participants in [28] and in six patients suffering from amyotrophic lateral sclerosis (ALS) in [29]. [30] showed in 12 users that frustration is related to BCI control. In [31] a strong correlation between mental imagery (including non-motor imagery tasks) BCI performance and mental rotation ability was found in an experiment with 18 users. In [32], MI-BCI performance was influenced by spatial ability, and difficult pre-training showed to improve participants’ capabilities of learning the BCI task.

More recent studies focus on modeling the neurophysiological mechanisms of BCI performance. The papers [17, 33–37] are based on the dataset presented in this manuscript and are discussed later. Regarding other studies, [38] showed that gamma activity in the fronto-parietal network is related to intra-subject trial-wise MI performance variations. Additionally, a weak negative correlation between centro-parietal gamma oscillation and the magnitude of the classification output was found. In a study with ten users, [39] found that prefrontal gamma band activity is positively correlated with MI performance in an inter-subject experiment, concluding that high prefrontal gamma activity, possibly related to the user’s concentration level, could be used as mental state to predict MI performance. In a study performed with 52 users, [40] found that high theta and low alpha power might indicate low MI performance.

Unfortunately, all these studies, except for the last one, presented at the most results from 20 users, while large scale studies would better cover the wide range of BCI potential users and allow more precise demographic and inter-subject investigation.

So far, three studies were conducted on a large population of BCI novices, but all of them were performed during BCI exhibitions, where the experimental conditions are noisy and not well controlled, and the experiment should be fast. In [14], results on SMR-based experiments with 99 users were reported, but the cause of poor performance for part of the population was not analyzed. The same authors performed a similar study with an ERP-based BCI and 100 novice users [41] and finally [42], presented a study with 106 subjects who participated in a SSVEP experiment during CeBIT 2008. In none of these studies the brain signals were analyzed to explain the drop-out[CS1] for some participants.

With the aim to investigate the BCI inefficiency for SMR-based BCIs and to understand how to deal with this problem, a large-scale screening study with 80 BCI novice participants was executed in collaboration with the University of Tübingen. The design of the BCI experiment was the classical BBCI one, with calibration and feedback sessions [10, 11] and a full electrode configuration (128 channels) was used. Additionally, motor imagery (MI) runs were accompanied by rest EEG recordings, motor observation (MO) and motor execution (ME) runs for comparison. Finally, a wide range of psychological tests was carried out, prior, during and after the BCI session.

The results of the psychological tests have been reported in [33]. In particular, better visuo-motor coordination and concentration on a task were significantly positively correlated with classification accuracy in a MI-based BCI system. This result was confirmed in [32] as reported above. Also, participants who felt confident with controlling a technical device performed better with the SMR-BCI. On the same data, a neurophysiological BCI performance predictor (the SMR-predictor) was built based on rest recordings and presented in [17]. In particular, it was shown that the estimated strength of the idle *μ*-rhythm in C3 and C4 EEG channels during the rest EEG recordings was significantly correlated to later BCI performance during feedback. A recent large scale study (with 160 users) confirmed this result [43, 44]. Similarly, [34] showed that spatio-temporal filters of resting state EEG are able to predict the BCI performance employing the data presented in this manuscript. In [35], long-range temporal correlations in the calibration recordings of this same dataset could predict the performance in the feedback recordings.

Twenty of the 80 participants underwent also fMRI recordings, as presented in [45, 46]. In [45], it was found that the number of activated voxels in the supplementary motor area (SMA) was greater for those with better MI performance and in [46] it was shown that structural brain traits can predict individual BCI performance.

Here, we present extensive analyses and statistics on the classification results, the SMR activity, the reactive frequencies and the limb (left hand, right hand or foot) preferences of the 80 participants. Based on the classification accuracy obtained in the calibration (Cb) and feedback (Fb) MI runs, 3 categories of BCI users were detected and all data analyzed and presented to highlight the difference in the SMR activity among these 3 group. EEG data have been interpreted in order to hypothesize the most common drawbacks BCI users might encounter during a BCI session. Finally, the SMR-predictor [17], was used to gain additional insights on the SMR activity of the different groups and obtain a more precise estimation of the percentage of BCI inefficiency, which we hypothesize to correspond to the percentage of users presenting a flat or almost flat rest EEG spectrum.

The manuscript is structured as follows. In the first Section[CS2] the entire study is described. After that, the categorization of BCI users is presented. Then, grand average results of standard EEG data analysis are shown, with a deep analysis on the SMR changes observable depending on the task (MO, ME and MI, for the three limb movement combinations). The offline and online performances for each run are reported and analyzed as well. Furthermore, the spectra at rest are investigated and a short overview of psychological predictors is given. Finally, discussion and conclusions are provided.

## Experimental setup

The study was approved by the Ethical Review Boards of the Medical Faculty, University of Tübingen and was designed together with the Institute of Medical Psychology and Behavioral Neurobiology of the University of Tübingen. The study consisted of two sessions per participant executed in two different days: a psychological test session on day 1 and a BCI session with short psychological tests on day 2. Between the two sessions a maximum of 7 days was allowed, to minimize the fluctuation in the psychological state of the participants but still allow for some flexibility in the appointment.

### Participants

A total of 80 healthy BCI-novices took part in the study: 39 men, 41 female, (age = 29.9±11.5y), age range was 17-65. Participants were required to have full contractual capability and no neurological disease, e.g., epilepsy. Each of them gave written informed consent after having been informed about the purpose of the study. Half of the experiments were recorded in Berlin and half in Tübingen; 75% of the subjects was younger than 30. Subjects were paid 8 *ϵ* per hour for the participation in the study. There was no special motivation for the participants to achieve good performance. Data sets of two participants were excluded because of technical problems.

### Day 1: Psychological test session

The psychological test-battery on day 1 lasted about 3 hours. It consisted of a *vividness of movement imagery questionnaire* [47], and *performance* and *personality* tests. The results regarding these psychological tests and their correlation with the SMR-BCI performance were reported in [33].

### Day 2: BCI session

The BCI session on day 2 lasted about 5 hours and consisted of 10 EEG recordings with psychological tests and concentration tests in between.

#### Hardware

During the BCI session the participants were sitting in a comfortable chair with arms lying relaxed on armrests, approximately 1 m away from a computer screen. For the recording, a cap with 128 Ag/AgCl electrodes (EasyCap, Brain Products, Munich, Germany) and two multi-channel EEG amplifiers (Brain Products, Munich, Germany) were used. Brain activity was recorded from 119 channels located according to the extended 10-10 system, [48–50]. Three electrodes were used to record the left and right horizontal electrooculogram (EOG) and the right vertical EOG. The remaining six electrodes were used to record the electromyogram (EMG) activity from the arms and the right leg (two electrodes per limb). The reference was positioned at the nasion. Electrode impedances were kept below 10 *kΩ*. Brain activity was sampled at 1000 Hz and band-pass filtered between 0.05 and 200 Hz. The EMG/EOG channels were exclusively used to control for physical limb/eye movements that could correlate with the task and could be reflected directly (artifacts) or indirectly (afferent signals from muscles and joint receptors) in the EEG channels.

#### EEG recordings

**Fig 1:**
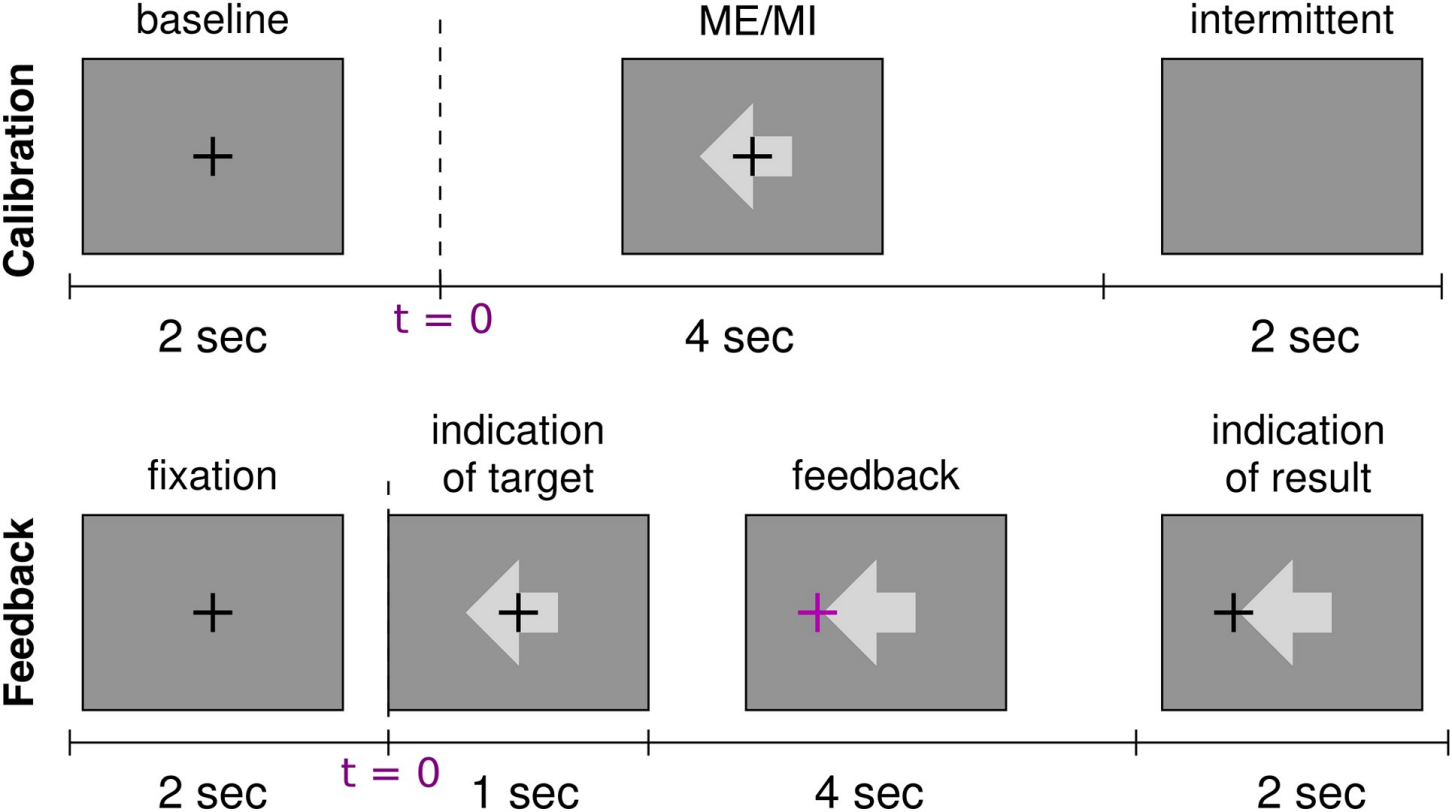
Trial design for calibration and feedback runs. First row: design of the trial for the ME and MI-Cb runs. The trial starts with a warning cue in the center of the screen in form of a cross at *t* = [−2 0]*s* (baseline or pre-stimulus interval). After 2*s* (i.e. at *t* = 0), the stimulus appears in form of an arrow. The direction of the arrow indicates the task to execute: left for left hand movement, right for right hand movement, down for foot movement. After 4*s*, cross and arrow disappear, and the screen stays blank for 2*s*. Then a new trial starts. One trial lasts therefore 8*s* in total. Second row: design of the trial for the MI-Fb runs. A fixation cross appears for 2*s* (baseline or pre-stimulus interval) at *t* = [−2 0]*s*. At *t* = 0 the stimulus appears. After 1*s*, the cross turns purple and starts to move depending on the classifier output (feedback). After 4*s* the cross turns black again and freezes for 2*s* while the result is visualized on the screen. One trial lasts therefore 9*s* in total.

EEG activity was recorded during the following 10 runs:

1. **Artifacts**. The user performed a sequence of tasks indicated by vocal instructions. The tasks were 1) maximum compression of the limbs, 2) looking to the right, left, center, top and bottom of the screen 3) blinking 4) relax with open and closed eyes. The duration of this recording was about 10 min.
2. **Motor Observation 1 (MO1)**. The subject simply watched video clips of 10*s* showing the movement of the left hand, right hand or foot from a first person’s perspective. They were presented in random order with 20 trials per class (*Left*, *Right*, *Foot*, where in the following *Left* and *Right* refer to left and right hand respectively). The participants were instructed to carefully observe and mentally imitate the observed movement from a first person’s perspective. The duration of this recording was about 20 min.
3. **Motor Execution (ME)**. The user chose the movement to imagine for the BCI session for the left hand, right hand and right foot, and really executed the motor task during this run. A stimulus in form of an arrow indicated the task to execute: left hand movement if the arrow was directed to the left, right hand movement if the arrow was directed to the right, right foot movement if the arrow was directed to the bottom.
  This run had 75 trials, 25 trials per class. Every 15 trials (one block) there was a break of about 20*s*. The order of the classes was random, but within each block the classes were equally distributed. The trial design is depicted on the first row of Fig 1. The duration of this recording was about 10*min*.
4. **Three runs of Motor Imagery Calibration (MI-Cb 1-3)**. The user did not execute the movement, but only imagined it kinesthetically [51]. The trial design was the same as for the ME run, illustrated on the first row of Fig 1. Also the number of trials per class and per run was the same as for the ME run. Thus, three MI-Cb runs provided a total of 225 trials, 75 per class (calibration data set).
  A short break (2 to 10 minutes depending on the subject’s needs) between the runs was used to let the participants fill in psychological tests. In total, the duration of the calibration recording was about 1 h.
5. **Three runs of Motor Imagery Feedback (MI-Fb 1-3)**. The subject imagined the movement kinesthetically as in the MI-Cb, but only for 2 out of the 3 motor tasks (left/right, left/foot or foot/right). In addition, his/her EEG activity was classified in real-time and a visual feedback was provided showing the classification results (a cross moving in one of the two possible directions).
  Each run consisted of 100 trials (50 trials per class). The feedback trial design was very similar to the calibration one and is depicted on the second row of Fig 1. Every 20 trials (one block) there was a break of about 20*s* where first the score (number of hits and misses summed over the blocks) and then a countdown starting from -15 were shown. The order of the classes was random, but within each block of 20 trials the classes were equally distributed (i.e. 10 trials per class in each block).
  The MI-Fb session lasted around 1*h* (each run lasted around 17*min*).
6. **Motor Observation 2 (MO2)**. This run was the same as MO1.

The MO runs at the beginning and at the end of the BCI sessions were included to investigate the SMR activity during MO and compare it to MI, as this was not extensively investigated in previous literature. The ME before the MI runs was mainly included to help the participants to get familiar with the task.

The number of trials of the MI-Cb runs was decided based on previous experience showing that a calibration data set of about 80 trials is necessary to train the machine learning BBCI algorithms.

The two out of three classes to perform in the MI-Fb runs were chosen based on the calibration data from the MI-Cb session (see Methods section for a detailed explanation).

Because of fatigue or lack of time, six participants completed only two runs and seven only one run, while the remaining 67 (83.75%) performed all three feedback runs.

Before each BCI run, instructions were provided on the screen so that all users received the same instructions. Furthermore, a short demo run was conducted. In the following, the course of the experiment is explained in detail.

#### Psychological questionnaires

During the preparation of the EEG cap, which took about 45 min, the participants were asked to fill in two questionnaires about their mood and motivation respectively. Additionally, after each MI run, the users answered a short questionnaire (run wise test) to describe the movement they imagined, their tiredness, motivation, angriness due to errors, uneasiness during the run, and how easy the imagination of movements was for them.

#### D2-test

Between the calibration runs of imagined movement, participants performed a computerized version of the d2-test [52], with the goal of reducing the monotony of the experiment.

## Methods

All methods applied in this work have been extensively used by the BBCI group and the code has been made public (https://github.com/bbci/bbci_public). In the following, methods used both during the BCI session and later for offline analysis are described. Proper EEG processing determines the success of a BCI and is an important topic in EEG analysis (see e.g. [50, 53–56]. Out of the 119 EEG channels, only those corresponding to the motor areas were used for parameter selection and classification purposes, while all 119 channels were used for scalp-plots visualization purposes.

### Automatic artifact rejection

The channels with impedance above 50 *kΩ* are removed. Afterwards, a simple variance-based artifact rejection is applied to reject trials and channels with evident amplitude abnormalities in the time interval between 500 ms and 4500 ms after the presentation of the visual stimulus. In particular, each trial is rejected with a standard deviation higher than twice the mean overall standard deviation and the procedure is iterated until no more trials are rejected. This method is applied for any of the results presented in this manuscript.

### Subject-specific frequency band and time interval selection

The subject-specific parameters to select are the reactive frequency band in which the spectra of the two classes are mostly discriminated, and the time interval in which the ERD of the two classes are mostly discriminated. This is done by an automatic procedure described in [57].

For the selection of the frequency band, the spectrum is calculated in the frequency range 5-35 Hz of the Laplacian derivation of the channels in the motor areas (peripheral channels are thus left out, as far as not specified) and averaged across trials of the same class. To asses how discriminative the spectrum is in each frequency bin, we used the signed *r*^2^-value (point biserial correlation coefficient, see corresponding section in Offlne analyses). It is calculated for each channel and each frequency bin separately because it can only deal with univariate features and then it is smoothed with a sliding window of 3 Hz. Briefly, the most discriminative frequency bins are chosen using heuristics, where the highest *r*^2^-value (across channels), and the lower and upper bound of the frequency band are iteratively enlarged until all frequency bins with the *r*^2^-value not lower than 1/3 of the initial highest *r*^2^-value are selected.

A similar procedure is applied for the selection of the time interval where the band-pass filtered and smoothed signals are mostly discriminated. In this case, the *r*^2^-values are calculated for each time point and each channel.

Typically, the data are first band-pass filtered in a large band (8-32 Hz). Then the time interval selection is applied. The data are then segmented using the selected time interval before applying the frequency band selection. Finally, the time interval selection is applied again with the data filtered in the reactive frequency band.

These parameters might be also manually selected by visually exploring the power spectrum and the ERD. During the BCI session, a semi-automatic selection was applied, i.e. the result of the heuristic selection was visually checked and confirmed or adjusted. This helped to avoid, for example, that the late time interval corresponding to a beta rebound is selected.

Especially when the classes are poorly discriminable consecutively for some frequency bins, the selection might fail choosing narrow time intervals and frequency bands. In this case a good strategy is to fix one of the two parameters, or to impose a minimum interval length.

### Laplacian derivation (LAP)

A Laplacian derivation [58, 59] of one channel is calculated subtracting the activity of *M* surrounding channels weighted by 1/*M* from the activity of the channel itself. Therefore, the Laplacian derivation weights all involved channels always in the same way, without considering the classes. It then results **W** = [1, −1/*M*, …, −1/*M*] and the new spatially filtered data are s^(t)=x0(t)−1M∑c=1Mxc(t), with *x*_0_(*t*) the data of the center channel of interest and *x*_*c*_(*t*) one of the surrounding *M* channels.

Clearly, this simple filter is not used to recover the brain sources, but mainly to eliminate background noise which is supposed to be present in all involved channels. This spatial filter is used in this work for the analysis of the EEG activity at channel level.

### Common Spatial Patterns (CSP)

Common Spatial Patterns (CSP) [60] is a discriminative algorithm which determines the spatial filters **W** from band-pass filtered EEG data such that the difference between the variances of the filtered data for the two classes is maximized.

This is done by a simultaneous diagonalization of the estimated covariance matrices $\Sigma_{1}=X_{1}X_{1}^{⊤}$ and $\Sigma_{2}=X_{2}X_{2}^{⊤}$ of the data for the two classes:

$$\begin{array}{ccc} W^{⊤}\Sigma_{1}W & = & \Lambda_{1} \end{array}$$ (1)

$$\begin{array}{ccc} W^{⊤}\Sigma_{2}W & = & \Lambda_{2}, \end{array}$$ (2)

$$\begin{array}{ccc} \text{s}.\text{t}.\;\Lambda_{1}+\Lambda_{2} & = & I \end{array}$$ (3)

where **Λ**_1_ and **Λ**_2_ are diagonal matrices and each λ on the diagonal corresponds to an eigenvector **w**^⊤^. In this way, the eigenvectors are the same for both decompositions and the same eigenvector, i.e. a spatial filter, corresponds to a large eigenvalue for one class and to a small eigenvalue for the other class. Since eigenvectors with large eigenvalues correspond to a large variance of the data, spatial filters with extreme eigenvalues maximize the difference in the variances for the two classes.

The sum of the formulas in Eq 3 forms the generalized eigenvalue problem:

$$\begin{array}{c} \Sigma_{2}W=\left(\Sigma_{1}+\Sigma_{2}\right)W\Lambda \end{array}$$ (4)

Choosing *D* filters corresponding to extreme eigenvalues (either close to 1 or close to 0) the filtered data $\hat{s}\left(t\right)=W_{D}^{⊤}X$ will have smaller dimensionality *D* < *N* and the two classes will be maximally separated by their variance.

A CSP feature, is the log-variance of the band-pass and CSP filtered data.

Two ways are employed in this thesis to choose the number of filters to use: 1) Three filters per class, for a total of 6 filters 2) Heuristic for the selection of an optimal number of filters, up to six.

The filter selection can be done either depending on the eigenvalues or by other scores which measure the discriminability between the data of the two classes: Area Under the Receiver Operating Characteristic (ROC) curve, indicated as AUC, the Fisher correlation coefficient and the *ratio-of-medians*. This last one is defined as:

$$\begin{array}{c} rms_{j}=\frac{m_{j,2}}{m_{j,2}+m_{j,1}} \end{array}$$ (5)

where *m*_*j*,1_ and *m*_*j*,2_ are the medians of the *j* − *th* CSP feature across all trials belonging to class 1 and 2 respectively. A score *rms*_*j*_ close to one indicates that the corresponding feature maximizes the variance for class 2 while a score close to zero indicates that the corresponding feature maximizes the variance for class 1. Choosing the features with an extreme score implies that the CSP feature of the two classes will be maximally separated. This *ratio-of-medians* score has been suggested in the CSP review [57] as being more robust with respect to outliers than the classical eigenvalue score.

### Classifiers

For the online system MI-Fb a Linear Discriminant Classifier (LDA) classifier was used since the EEG preprocessing was done semi-automatically, i.e. the experimenter visually checked the data (spectra, ERD and CSP filters) and ensured that no overfitting was occurring. For offline analysis, all algorithms were automatic and the use of a Linear Discriminant Analysis (LDA) with shrinkage was preferred, as it is more robust against overfitting. LDA finds a one-dimensional subspace where the classes are well-separated. This is formalized by maximizing the ratio of the between-class variance to the within-class variance after the projecting onto the subspace. For two classes the optimal subspace is defined by

$$\begin{array}{ccc} w & = & \Sigma^{-1}(\mu_{1}-\mu_{2})\;, \end{array}$$ (6)

where **Σ** is the sample-covariance matrix, and *μ*_1_, *μ*_2_ are the sample class means. As the covariance matrix is often typically poorly conditioned, we can follow the approach by Ledoit & Wolf [61] and replace **Σ** in Eq (6) by a shrinkage estimate of the form

$$\begin{array}{ccc} \Sigma_{\lambda } & = & (1-\lambda )\Sigma +\lambda \tilde{\Sigma },\;\lambda \in \left[0,1\right]\;. \end{array}$$

The matrix $\tilde{\Sigma }$ is the sample covariance matrix of a restricted sub-model, and the optimal shrinkage intensity λ can be analytically estimated from the data. We use the following sub-model: all variances (i.e. all diagonal elements) are equal, and all covariances (i.e. all off-diagonal elements) are zero.

### BBCI machine learning algorithm training

During the BCI session, the collected MI-Cb data were used to train the machine learning algorithms according to the BBCI advanced machine learning procedure, [10, 11, 57, 62]. The algorithm training consists of the following steps:

1. Automatic artifact rejection

Per each of the three class combinations (*Left/Right*, *Left/Foot* and *Foot/Right*:

2. Semi-automatic selection of the subject-specific frequency band and time interval where the classes are best discriminated.
3. Band-pass filtering data and segmentation using the subject-specific frequency band and time interval.
4. Semi-automatic selection of up to six subject-specific CSP filters (up to three per class).
5. Extraction of CSP features: the log-variance (i.e. the logarithmic band power) of the band-pass filtered, segmented and CSP spatially filtered data.
6. Calculation by 8-fold cross-validation (CV) of the *global error* of a LDA classifier trained on the CSP features.

In this way, overfitting may occur, because the CSP has been trained on the whole dataset (outside the CV). For the selection of the best class combination, a *generalization error* (or *calibration error* is additionally estimated by 8-fold CV with the extraction of CSP features (and LDA classification) per each fold.

The class combination with the best generalization error was selected to be used in the MI-Fb runs.

**Fig 2:**
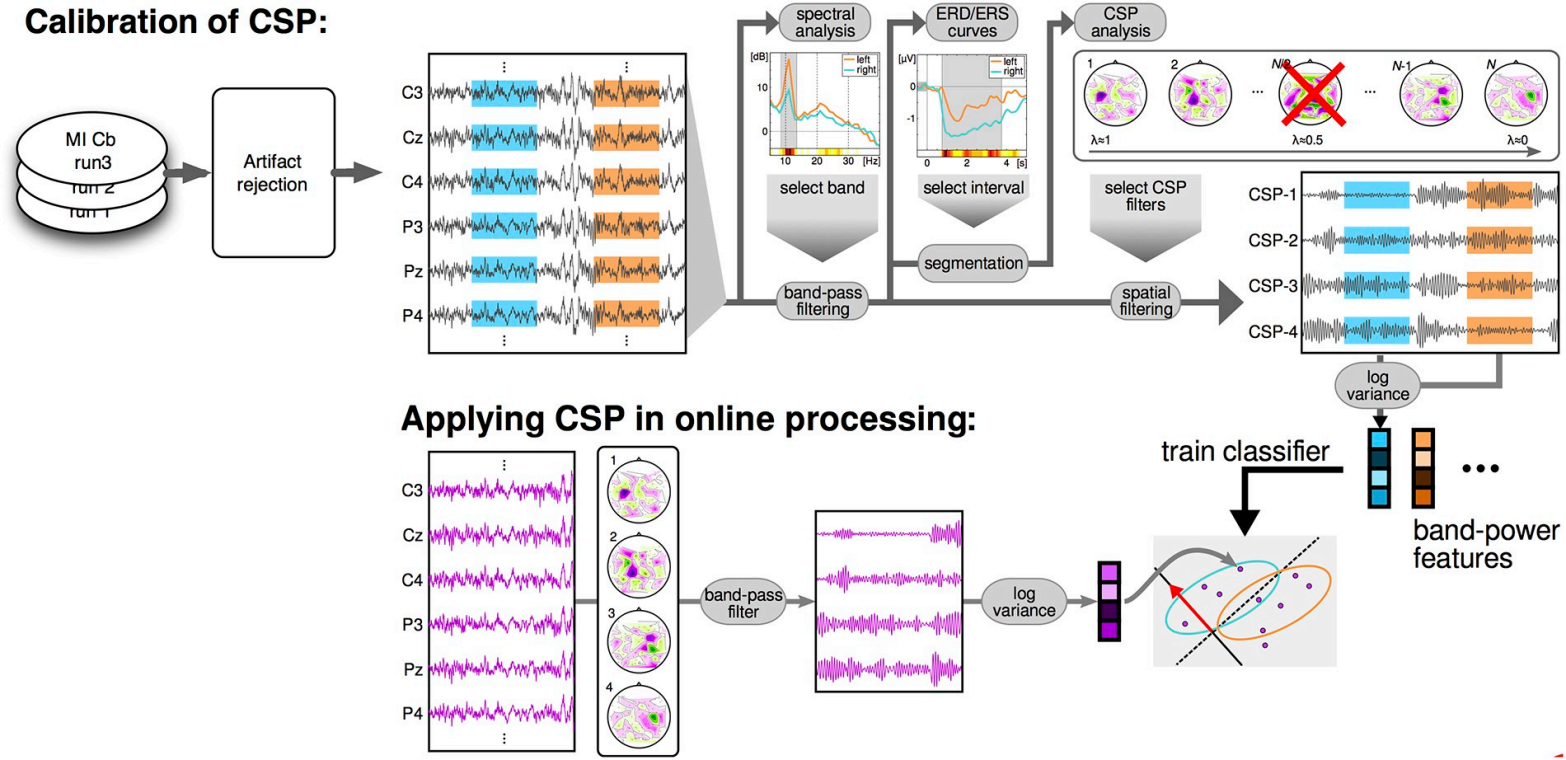
Dataflow of the processing applied during the BCI session. Once the MI-Cb data are collected, artifact rejection and heuristic for the selection of subject-specific frequency band and time interval are applied. CSP filters are then calculated by cross-validation, and a linear classifier is trained on the log-variance features of the CSP filtered data. The same frequency band, CSP filters and classifier, are then applied in real-time during the feedback session to provide the visual feedback to the user. The classification is applied during the whole trial, i.e. the subject-specific time interval is not used during the online feedback.

A flowchart of the data analysis applied for the BCI session is shown in Fig 2.

### Offline analyses

For the offline analysis, a representative SMR-channel is often used to investigate the SMR activity of the single user. The SMR-channel is the electrode with the highest squared point biserial correlation coefficient (*r*^2^-value). It is a correlation coefficient between a real variable (in this case the band power in the subject specific band) and a dichotomous one containing class information. It is used as discriminative measure for univariate features. Values close to 1 indicate that the feature is very correlated to the class, i.e., it is discriminative. Values close to 0 mean that the feature is not discriminative for the two classes.

$$r^{2}\left(x,y\right):=\frac{N_{1}\cdot N_{2}}{{\left(N_{1}+N_{2}\right)}^{2}}\;\frac{{\left(\mu_{1}-\mu_{2}\right)}^{2}}{\mathrm{var}\left\langle x_{i}\right\rangle }$$

with $\mu_{1}=\text{mean}{\langle x_{i}\rangle }_{y_{i}=1}\;\text{y}\;\mu_{2}=\text{mean}{\langle x_{i}\rangle }_{y_{i}=2}$ the mean values of the classes and *N*_*k*_ = |{*i*|*y*_*i*_ = *k*}| the number of observations of each class *k*. The signed *r*^2^ is obtained multiplying *r*^2^ with the sign of ***μ***_1_ − ***μ***_2_. The signed *r*^2^-values are not normally distributed and therefore not indicated in case a grand average is needed among different subjects. In such cases, a *z*-score transformation is applied to the *r*^2^-values of each subject prior to averaging:

$$\begin{array}{c} z=arctanh\left(r\right)=ln(\sqrt{\frac{1+r}{1-r}}) \end{array}$$ (7)

The result are normally distributed *z*-scores. Thus, a *p*-value can be therefore assessed on the grand-average (p ≤ 0.05, which corresponds to |z| ≥ *log*(0.05) ≃ 3).

**Fig 3:**
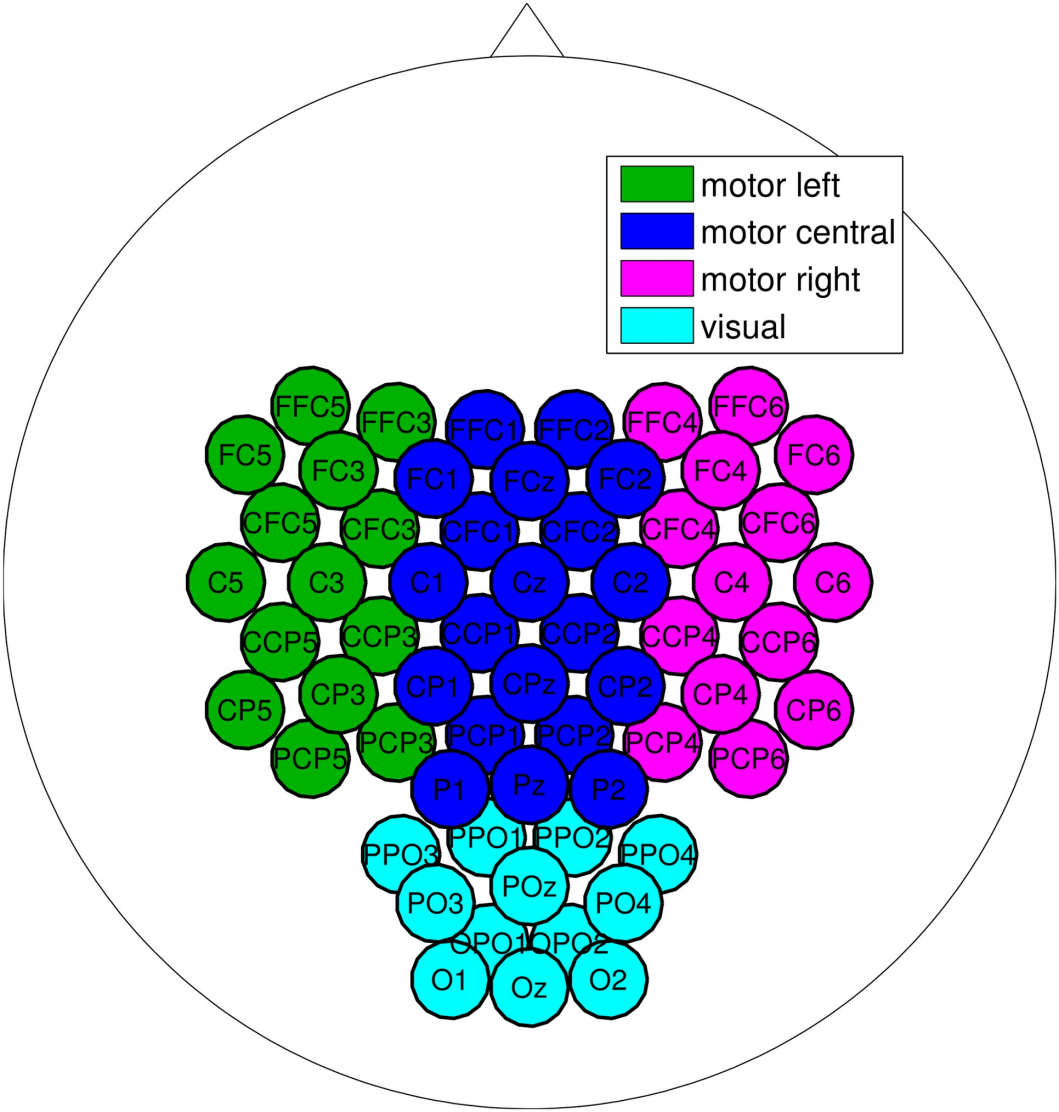
Channels used to select the *SMR-channel*.

To select channels, derivations from the left, central or right sensorimotor area were chosen. Additionally, for the MO runs, the channels from the parieto-occipital area were also candidates as *SMR-channel*. Fig 3 depicts the channels used for the selection.

For the offline grand average analysis, subject-specific frequency and time intervals for each MO run, for the ME run and for the three MI-Fb runs were re-calculated using the corresponding trials, while the parameters used during the experiment were selected using the MI-Cb runs. This allows to analyze the time course of the SMR activity from run to run. Since the MO and ME runs had just respectively 40 and 50 trials (considering the two selected classes), the expected Signal to Noise Ratio (SNR) was not optimal, and the parameter selection was done in the semi-automatic way. For the three MI-Fb runs, the selection was automatic and was done in order to assess how stable the SMR activity was during the MI tasks and passing from calibration to feedback.

The offline CSP analysis was conducted on the band-pass filtered and epoched data shown in Fig 2 and a Linear Discriminant classifier (LDA) corrected by shrinkage ([62–64]) was used to classify the resulting CSP features, i.e. different from the LSR classifier used online. This shinkage-LDA was used since all algorithms were run automatically and the LDA with shrinkage which is more robust against overfitting. The shrinkage is not possible with the LSR.

The offline classification accuracy was calculated as the area under the ROC curve (AUC), were ROC stands for Receiver Operating Characteristic. This was made twice as follows:

1. with subject-specific frequency and time interval, CSP filters and LDA used during the experiment, i.e. chosen from the three MI-Cb runs. In this case the data from each run are then the test set, where the classification accuracy is calculated. To avoid overfitting, for each MI-Cb run, CSPs and LDA are re-calculated using the other two runs as training set. This accuracy is called *transfer* accuracy, since it measures the transferability of parameters, spatial filters and classifier from MI-Cb runs to the other runs.
2. with subject-specific frequency and time interval chosen on the same run. In this case, CSP filters, LDA and classification accuracy are calculated by cross-validation (CV). To take into account the disparity of number of trials in the training set, leave-one-out CV (LOO-CV) was used for the MO, ME and MI-Cb runs, which had respectively 40, 50 and 50 trials, while 2-fold CV was used for the MI-Fb runs, which had 100 trials each. This accuracy is called *inside* accuracy.

### Neurophysiological predictor from spectra at rest

The EEG power spectrum at rest in the motor areas can be described by a 1/*f* curve, where *f* is the frequency, with one, two or (rarely) three peaks around 10, 20 and 30 Hz and motor imagery causes a suppression of these peaks by desynchronization of the underlying cortical networks. The 1/*f* curve indicates the EEG background activity, which is called noise because the higher it is, the more the EEG rhythmic activity of interest, i.e. the peaks, is hidden. It can then be assumed, that users with more prominent peaks in the spectrum at rest have a higher potentiality to suppress them. Additionally, not just the absolute peak amplitude, but the level of noise is important as well, which should be as low as possible.

Based on this neurophysiological assumption, in [17] a SMR-predictor is presented, which allows to predict with high reliability how likely a subject can achieve BCI control by SMR modulation. To calculate the SMR-predictor, the distance between the Power Spectrum Density (PSD) at rest and the noise curve at a particular scalp location is estimated by modeling both the PSD and the noise curve. In fact, the maximal distance between the peaks in *α* and *β* and the noise for each channel can be considered as the *SMR-strength* over that channel location. In the following section, a re-formulation of the model presented in [17] is separately described, which is good in general as model for the EEG spectrum. The model itself was developed in [17]. Here, the model is used to analyze the EEG data at rest of subjects in each category. The model of the EEG PSD curve is constructed as the sum of three functions *n*, *g*_*α*_ and *g*_*β*_ modeling respectively the noise, the peak in the *α* frequency band and the peak in the *β* frequency band. The noise is modeled by a hyperbolic function, while the two peaks are modeled by Gaussian functions *φ*. It results the following function $\hat{PSD}$ of frequency *f*:

$$\begin{array}{ccc} \hat{PSD}\left(f;\lambda ,\mu ,\sigma ,k\right) & = & n\left(f;\lambda ,k_{n}\right)+g_{\alpha }\left(f;\mu_{\alpha },\sigma_{\alpha }\right)+g_{\beta }\left(f;\mu_{\beta },\sigma_{\beta }\right)\text{,} \end{array}$$ (8)

$$\begin{array}{ccc} n(f;\lambda ,k_{n}) & = & k_{n1}+\frac{k_{n2}}{f^{\lambda }}\text{,} \end{array}$$ (9)

$$\begin{array}{ccc} g_{\alpha }\left(f;\mu_{\alpha },\sigma_{\alpha }\right) & = & k_{\alpha }\varphi \left(f;\mu_{\alpha },\sigma_{\alpha }\right)\text{,} \end{array}$$ (10)

$$\begin{array}{ccc} g_{\beta }\left(f;\mu_{\beta },\sigma_{\beta }\right) & = & k_{\beta }\varphi \left(f;\mu_{\beta },\sigma_{\beta }\right) \end{array}$$ (11)

The parameters λ and *k*_*n*2_ regulate the shape of the noise function *n*, while *k*_*n*1_ regulates its amplitude. The parameters *k*_*α*_, *μ*_*α*_ and *σ*_*α*_, regulate respectively the amplitude, the position and the width of the Gaussian function *g*_*α*_ representing the peak in *α*. The peak in *β* is also modeled by its own parameters. The function $\hat{PSD}$ is thus modeled in total by nine parameters (in Eq 11 indicated by λ, *μ* = (*μ_α_*, *μ_β_*), *σ* = (*σ_α_*, *σ_β_*) and **k** = (*k*_*n*1_, *k*_*n*2_, *k*_*α*_, *k_β_*), all ∈R). As objective function for the optimization of the nine parameters, the *L*_2_ − *norm* of the difference vector $PSD\left(f\right)-\hat{PSD}\left(f;\lambda ,\mu ,\sigma ,k\right)$ is taken, where f is the frequency vector with *f* in the range 2-35 Hz.

## Results

### Categorization of BCI users

In order to analyze the data with a special focus on participants who had difficulties in achieving the BCI control, users were categorized depending on their SMR activity and performance during the MI runs. A threshold criterion of 70% was used, to assess the three main categories, called I, II and III. This performance level for binary BCIs used for communication purposes was established in [26], observing that just above this threshold BCI users feel to be able to control the machine. Additionally, subcategories were found, by inspection of the SMR activity of all users in the MI-Cb and MI-Fb sessions as described in the next sections.

#### Calibration vs. feedback data comparison

**Fig 4:**
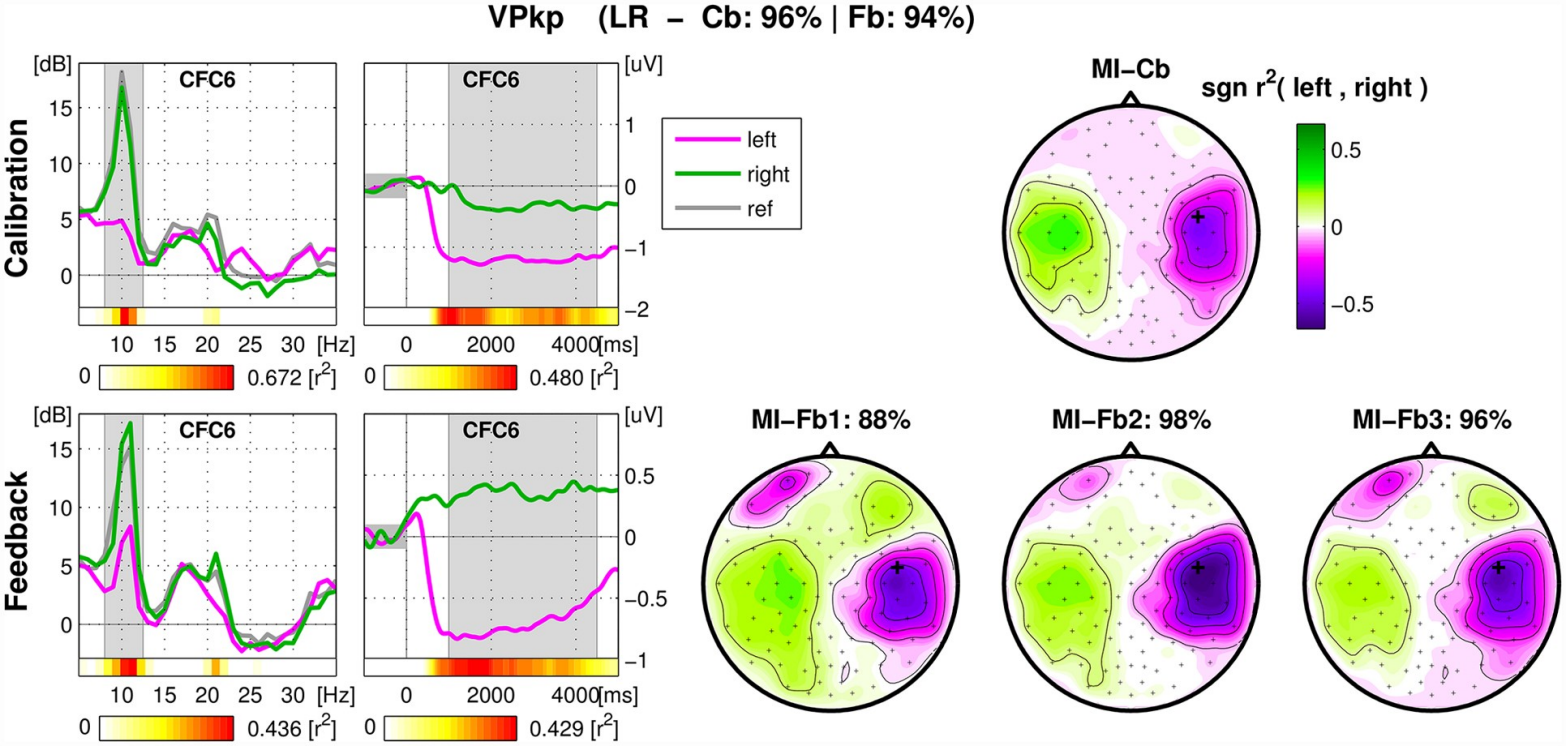
Overview figure of calibration and feedback runs for one user. The title of the figure contains the classes chosen for the feedback (LR in case of *Left/Right* otherwise LF for *Left/Foot*, FR for *Foot/Right*), the calibration and the feedback performance. Left, first column: spectrum (dB vs. Hz) of the SMR-channel averaged across trials of the same class (magenta for *Left*, green for *Right*). In gray, the spectrum calculated in the 1000 ms pre-stimulus. The subject-specific frequency band chosen during the experiment is marked in gray. For each frequency bin the color coded *r*^2^-values are depicted in the horizontal bar below the spectrum. The second external horizontal bar indicates the corresponding scale. Left, second column: ERD of the same channel (μV vs. Hz), calculated in the frequency band marked in the spectrum plot and averaged across trials belonging to the same class. The time interval used to calculate the spectrum is marked in gray. Again, two horizontal bars indicate respectively the *r*^2^-values for each time bin and the corresponding scale. Right: signed *r*^2^-values plotted on the scalp with corresponding color scale. Top: calibration data. One scalp plot for 150 trials. Calibration and average feedback performance are indicated in the main title. Bottom: feedback data. One scalp plot per run with the corresponding feedback performance.

For each user, an overview figure was inspected, containing spectrum and ERD of the SMR-channel and signed *r*^2^ − *values* of all channels (scalp-plot) for MI-Cb and MI-Fb trials separately for direct comparison. An example of overview figure for a good performing user is shown in in Fig 4: the SMR modulation is strong for both classes *Left* hand and *Right* hand. In the case of this user, the scalp maps of the feedback runs display not only ERD/S on the sensorimotor areas, but also horizontal eye movements. This happens because during feedback some users moved their eyes following the cursor. When the performance is good, eye movements are correlated with the target and therefore appear in the signed-*r*^2^ plot. However, since the classifier was not trained on this effect, it does not affect the performance. Investigating these figures, it was possible to assess to what extent the algorithms trained on the calibration data are transferable on the feedback data and to hypothesize the causes of a performance drop.

#### Categories

Each category is divided in subcategories indicated by letters (a to c) as described below. User categorization can be then summarized as follows:

- **Category I**: Calibration Performance ≥ 70% and Feedback Performance ≥ 70%
  1. Strong modulation of SMR for at least 1 class and average feedback performance above 90%.
  2. Medium modulation of SMR for at least 1 class and average feedback performance below 90% but similar to calibration.
  3. Feedback performance weaker than expected from calibration data.
- **Category II**: Calibration Performance ≥ 70% and Feedback Performance < 70%
  1. Strongest SMR modulation at parietal area in calibration.
  2. Others: development of different patterns in the MI-Fb due to the visual feedback, timing problems in the ERD/ERS, tiredness and lack of concentration, difficulty in ignoring the visual cue during feedback (see text for detailed description).
- **Category III**: Calibration Performance < 70%, no feedback control possible.
  1. Weak SMR idle rhythm, weak SMR modulation
  2. Weak SMR idle rhythm, no class specific modulation.
  3. Almost no SMR idle rhythm, or not at all.

The difference between group Ia and group Ib is mainly given by their average performance, whether it is above or under 90%. Both groups exhibit similar estimated calibration performance and feedback accuracy. Cat. Ic includes people whose performance had a drop of about 10% between MI-Cb and MI-Fb sessions, with feedback performance still above 70%. Cat. IIa and IIb also presented a drop in the performance, but additionally they could not achieve control in the MI-Fb session (performance < 70%).

Users belonging to Cat. IIa presented a class related modulation in the parietal area. It can be hypothesized that these users imagined the movement more visually than kinesthetically since the patterns are similar to those reported in [51]. Indeed, poor feedback control could be expected, since the visual MI is known to be less classifiable than kinesthetic MI, as assessed in [51]. Moreover, the visual feedback processing might interfere with the visual MI.

**Fig 5:**
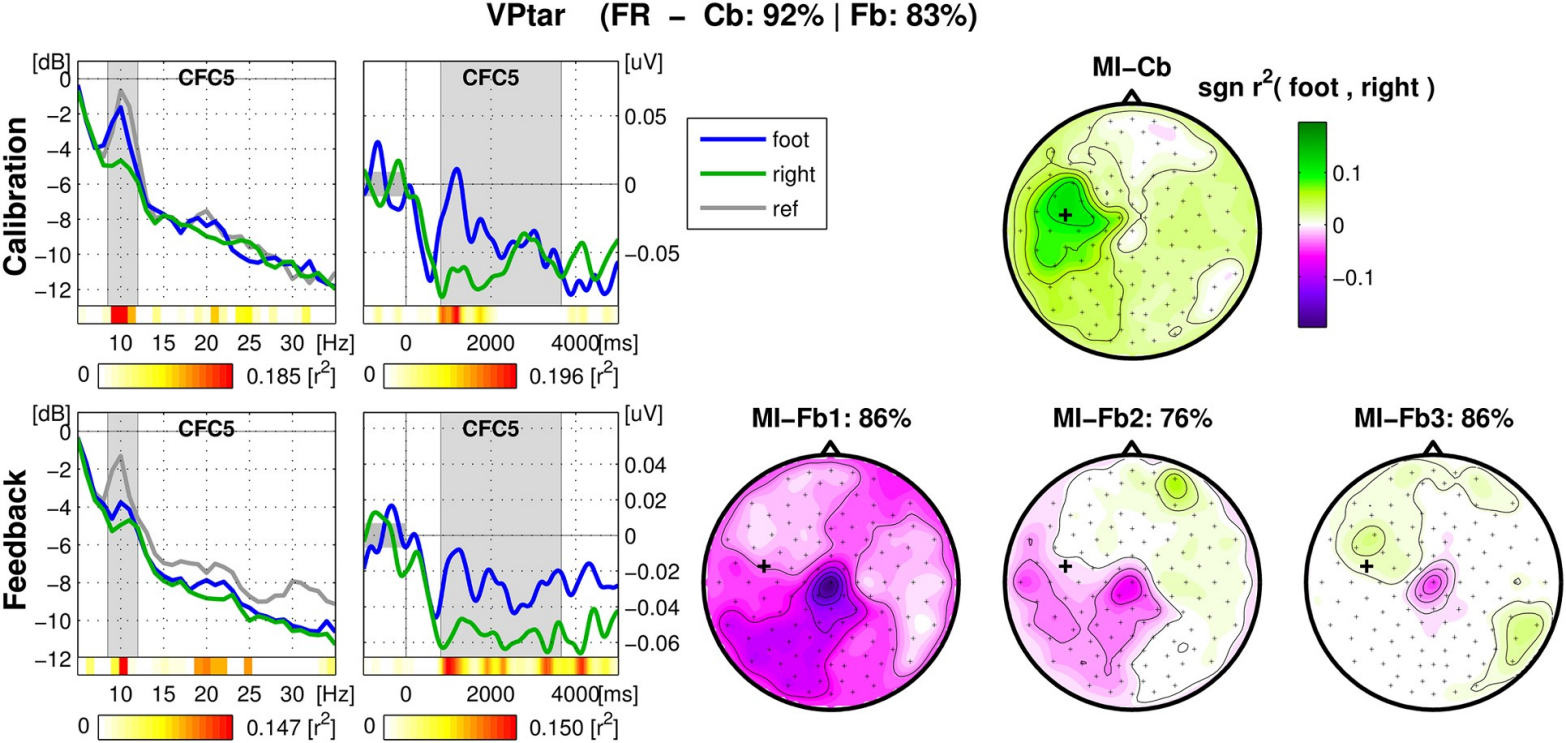
Overview figure of calibration and feedback runs for an exemplary user. Example of development of correct foot SMR pattern during MI-Fb session and of ERD for both classes prior to a resynchronization for the class *Foot* class. Also, it is visible how the feedback helped to maintain the ERD longer. From left to right, spectra, ERD and scalp plot of r^2^-values. Top: calibration data. Bottom: feedback data. See caption of Fig 4 for details.

By inspection of each figure the following hypotheses can be formulated for the performance drop in Cat. Ic and Cat. IIb:

1. Change of SMR patterns from the MI-Cb to the MI-Fb session. In these cases, the feedback influences the users positively, letting them develop new and proper SMR patterns which were not present during the MI-Cb session. Often, the user calibration data presented a good ERD for one class, proper (i.e. on the expected motor area) or not proper (more over the parietal or premotor area), that let train the algorithms and obtain a significant calibration performance. Thanks to the feedback, or sometimes because the user was not able to continue to imagine the same movement, during the feedback session the proper pattern of the other class appeared, mostly in the same frequency band. In some cases, this pattern started to appear already during the MI-Cb session and was caught from the CSP filters, so that it was possible to classify it in the feedback session. In most of cases, the pattern is completely new and led to a performance drop. In the worst cases, the new patterns developed in another frequency band, which was often the case for the foot synchronization pattern in the beta range and was not classifiable at all. In Fig 5, an example of a user is depicted who developed a foot ERD still weakly maintaining the right (a bit premotor) pattern which was much stronger during the MI-Cb session, (color blue is used for *Foot*).
  At least 11 users (13.75%) were found to change or develop their SMR rhythms during the feedback session, 4 were in the Cat. Ic, 7 in the Cat. IIb. All of them, except for one, reported in the run wise tests that they did not change MI strategy or movement.
2. Timing problems. For some users, the ERD plots presented a strong desynchronization (17 users) or synchronization (7 users) for both classes after the cue, before the feedback started. While these ERD/ERS might be caused by a particular MI strategy (sudden preparation with both limbs or strongly relaxation of both limbs before starting the MI), they imply either a partial ERD in the ipsilateral brain area (in case of ERD after the cue) or a late ERD in the contralateral brain area (in case of ERS after cue). Another timing problem, which affects the performance even more, is a short ERD. In fact, some users (at least 6) had too short ERD (2*s* on average), so that in many trials they could control the cross just shortly, while the classification continued to be assessed until the end of the trial, being the score assessed according to the final position of the cursor. Since the offline calibration performance and the trained algorithms refer to the subject-specific time interval, the ERD might be not long enough to obtain a correct online classification of the trial. Note that often the feedback helps the user to maintain the ERD longer or even till the end of the trial.
  Fig 5 is also a good example of timing problems. In fact, a short ERD characterizes the MI-CB data, while the feedback helped in holding the ERD longer. Still, both classes exhibit a sudden desynchronization after the stimulus presentation, so that during the feedback session the cortical networks under CFC5 stay desynchronized also during the foot imagination.
3. Tiredness or decrease of concentration. This could be the problem for those users (five in total among Cat. Ic and IIb) who kept the same SMR patterns as in the MI-Cb session, but less consistently, resulting in lower *r*^2^-values and worse performance.
4. Some users (at least two) encountered problems ignoring the visual cue, so that the feedback data contained head movements.
5. For two users the classification of calibration data was based on beta rebound, which was not stable enough to obtain good feedback performance.
6. Some users (at least two) misunderstood the task or changed completely the MI strategy, confirmation of this hypothesis was found in the run wise questionnaires.

Just one subject had calibration performance < 70% and feedback performance ≥ 70% and was classified in Cat. IIb. He had a more parietal SMR and did just one feedback run.

Table 1 shows the overview of the percentage of users for each category, together with their class preferences, calibration and feedback performance obtained during the experiment. These are the BCI experiment results and provide the statistics on the BCI performance of the general population, never published before with such accuracy on a large dataset.

**Table 1:** User categorization overview.

Cat.	a	b	c	N	LR	LF	FR	L	R	F	Cb-Perf. [%]	Fb-Perf. [%]
I	22	16	10	48 (60.0%)	23	19	6	42	29	25	90.99 ± 6.16	85.60 ± 9.94
II	5	9		14 (17.5%)	2	7	5	9	7	12	81.22 ± 8.24	61.79 ± 8.60
III	6	5	7	18 (22.5%)	5	8	5	13	10	13	64.46 ± 6.64	54.40 ± 4.97
					30	34	16	64	46	50	83.31 ± 12.68	74.42 ± 16.48

User categorization overview with corresponding percentage, class preferences, calibration and feedback performance. The first three columns refer to the subcategories (a, b and c if present), the fourth column is the sum of the first three, i.e. the total number N of users belonging to the corresponding category and the percentage in the population of 80 users. In the subsequent six columns, the number of users of each category is divided by the class combination they performed in the MI-Fb session (LR, LF, and FR) and by the single class used (L, R and F). Mean and standard deviation of calibration and feedback performance is reported in the last two columns.

It can be seen that 60% of the users achieved BCI control (Cat. I) with an average performance of 85%, 17.5% showed SMR modulation in calibration but failed in achieving BCI control in the MI-Fb session (Cat. II) and 22.5% did not exhibit an SMR modulation strong enough to calculate stable CSP filters already in the MI-Cb session (Cat. III). Note that just 7 participants, i.e. 8.75%, did not exhibit any, or almost any, idle SMR rhythm.

It can be also observed that most of the good performing users (actually all users of Cat. Ia except for 4) used the combination *Left/Right* hand. In fact, the use of the class *Foot* comes into play just when the desynchronization between left and right hemisphere results difficult. Actually, among Cat. II and III, most participants showed prevalence either for *Left/Foot* or for *Foot/Right*. Another evidence is that the class combination *Left/Foot* was also in general selected in comparison to *Foot/Right*, so that *Left* is the most often occurring class.

### Grand average analyses

Grand average analyses of the 80 dataset have been conducted in order to further investigate the three categories. In fact, given large number of data sets, single subject results are difficult to report. Nevertheless, given the high variability among the SMR activity of each user, especially in the Cat. II and III, parameters have been optimized on a single-subject level, before averaging the results by category and by class combination.

#### Reactive frequency bands

**Fig 6:**
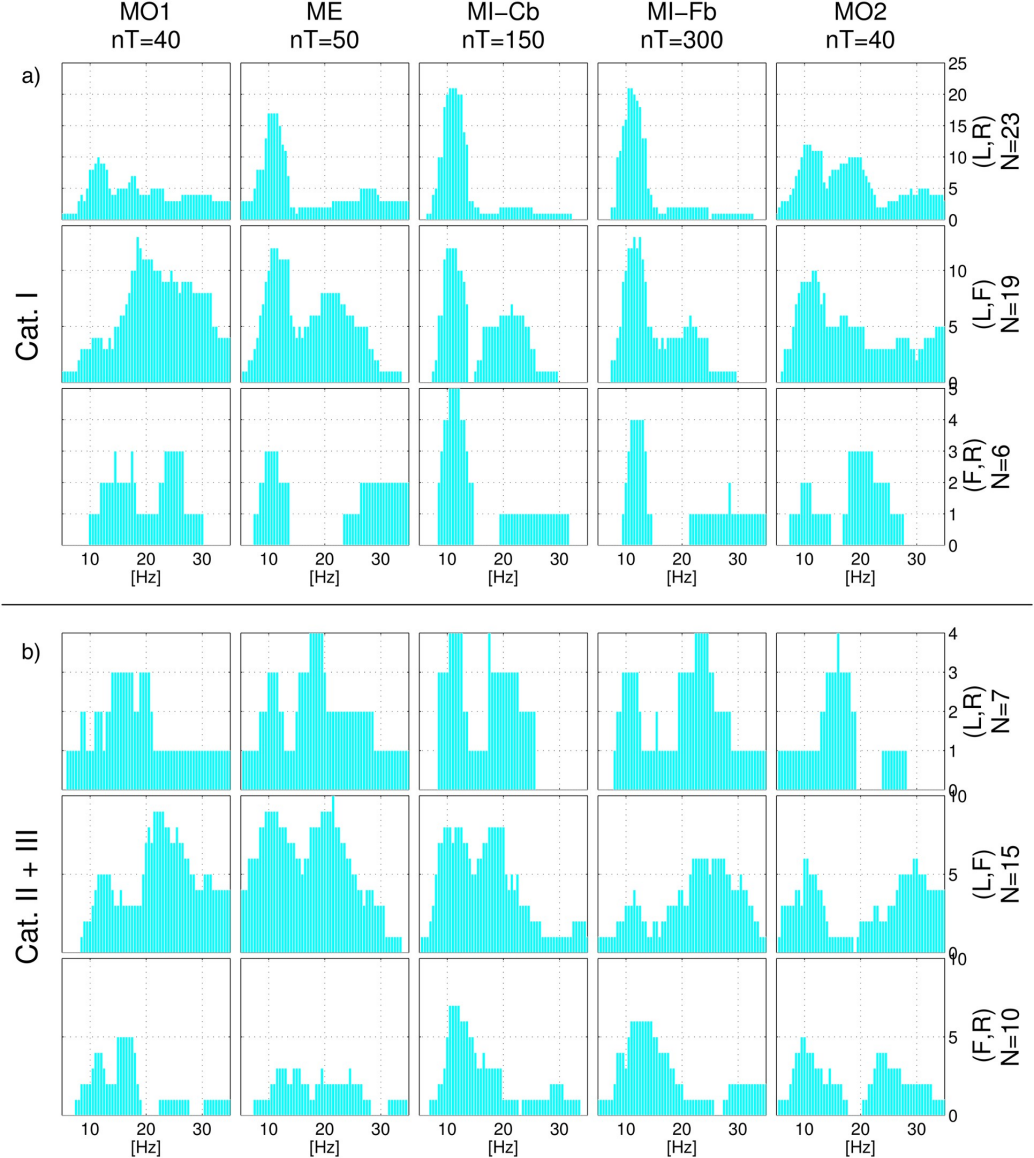
Histogram of reactive frequency bands. For each frequency bin, the histogram indicates the number of users for whom the frequency belonged to the subject-specific frequency band. From left to right, the different tasks/runs. Panel a) corresponds to Cat. I users and panel b) to Cat. II and III participants. In each panel from top to bottom, the three class combinations *Left/Right*, *Left/Foot* and *Foot/Right*.

Fig 6 shows histograms of the frequency selection of each frequency bin obtained calculating the subject-specific reactive frequency band in each single run. Panel a) refers to Cat. I users and can thus be considered as exemplary. The histogram of the first MO run (MO1) looks very different from the histograms of the other runs, where a peak in the *μ* range is evident. On the contrary, the ME run histogram is very similar to the MI-Cb and MI-Fb ones, where the *μ* peak around 12 Hz becomes even sharper. However, ME exhibits more *β* discriminative activity in comparison to MI. Conversely, for MI the *μ* band was more often selected than for ME. In the second MO run (MO2), peaks on *μ* and *β* appear, indicating the influence of the MI tasks on the developing of more stable SMR.

The frequency bands automatically selected on the active frequencies for the MI-Fb data are almost the same as those chosen for the MI-Cb data during the experiment, as expected for Cat. I users. It can also be observed, that for the *Left/Right* class combination 21 out of 23 users employed the *μ* rhythm to obtain BCI control, while a peak in the *β* range appears just when the *Foot* class comes into play. In particular, the inspection of the signed *r*^2^ scalp maps confirmed that, among users who used the *Left/Foot* or *Foot/Right* class combinations, just those who obtained BCI control mainly by the *Foot* pattern employed the *β* SMR, while most of the users obtained BCI control by the *Left* or *Right* pattern in the *μ* SMR.

Panel b) of Fig 6 depicts the frequency distribution for the user Cat. II and III (together because the number of users per class combination is low, see Table 1). Here, for some users the chosen reactive frequency bands changes from calibration to feedback, indicating a reason for poor feedback performance. Usually users developed during feedback new, often standard, SMR patterns. Differently from the frequency distribution for Cat. I users, it can be observed that the *β* band is employed much more frequently, also with the class combination *Left/Right*. Moreover, this happens also for the ME and MI-Cb runs, i.e. this phenomenon is not related to a deficient BCI control during feedback.

**Fig 7:**
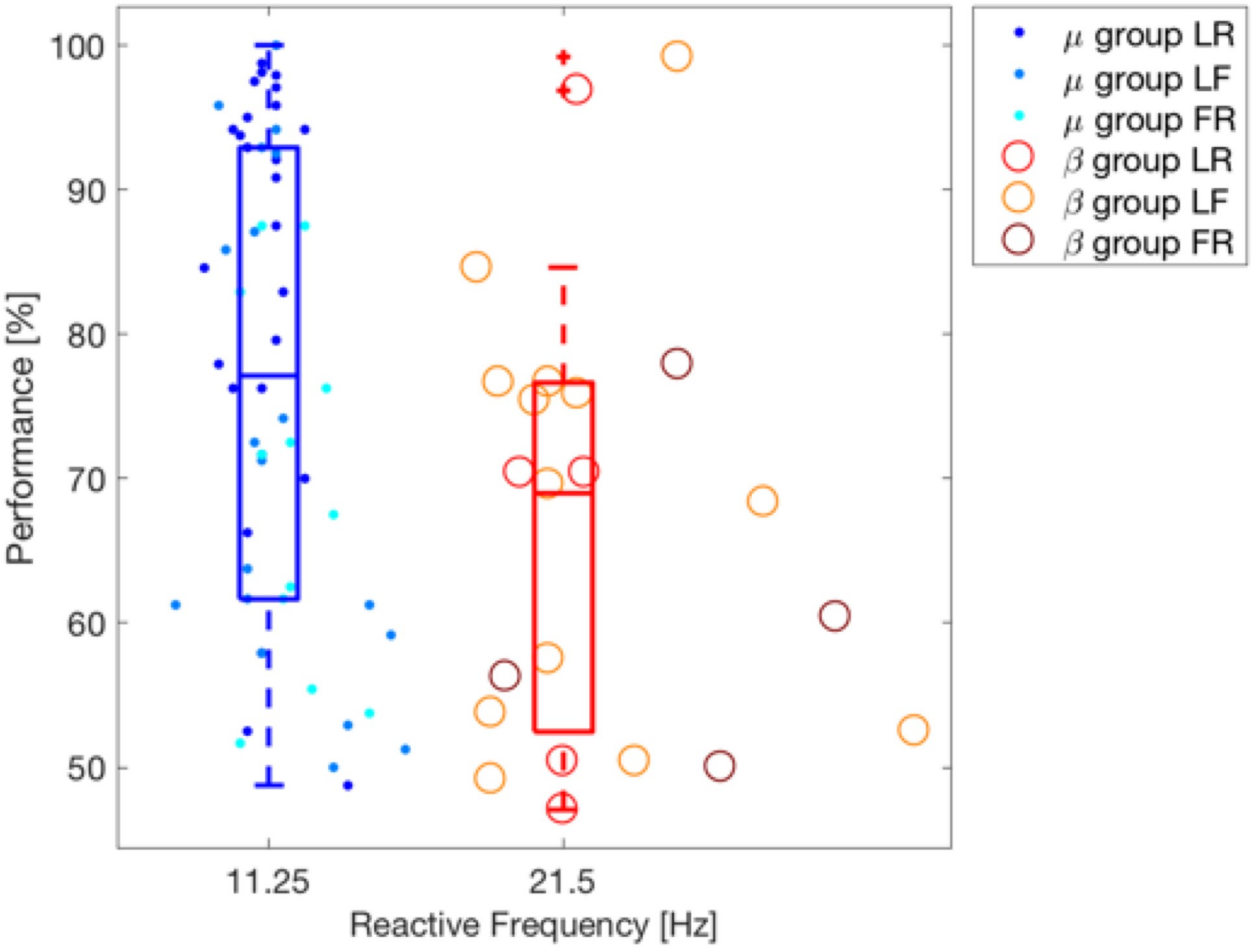
Clustering of users depending on their SMR reactive frequency band. K-mean clustering is applied on the values of the reactive frequency bands. One dot per subject, dots or circles depending on the cluster the user belongs to. Different tonalities indicate different class-combination for each of the two clusters. Feedback performance is shown on the y-axis. The box plots corresponding to the performance information for each cluster are also shown.

To test the hypothesis that higher frequencies are more reactive for motor imagery in poor performing users, we performed the clustering of users according with their reactive band using k-means with two clusters [65]. After that, we tested whether the performance of both groups significantly differed using a Wilcoxon test (one-tailed). The selected clusters correspond to the *μ* (11.25 Hz) and *β* (21.5 Hz) bands and the classification accuracy on the *μ* band (median 77.08%) is significantly better than that of the *β* band (median 68.96%) with p-value = 0.006. The result of this analysis is depicted in Fig 7.

#### Grand average data: MO vs. ME vs. MI

**Fig 8:**
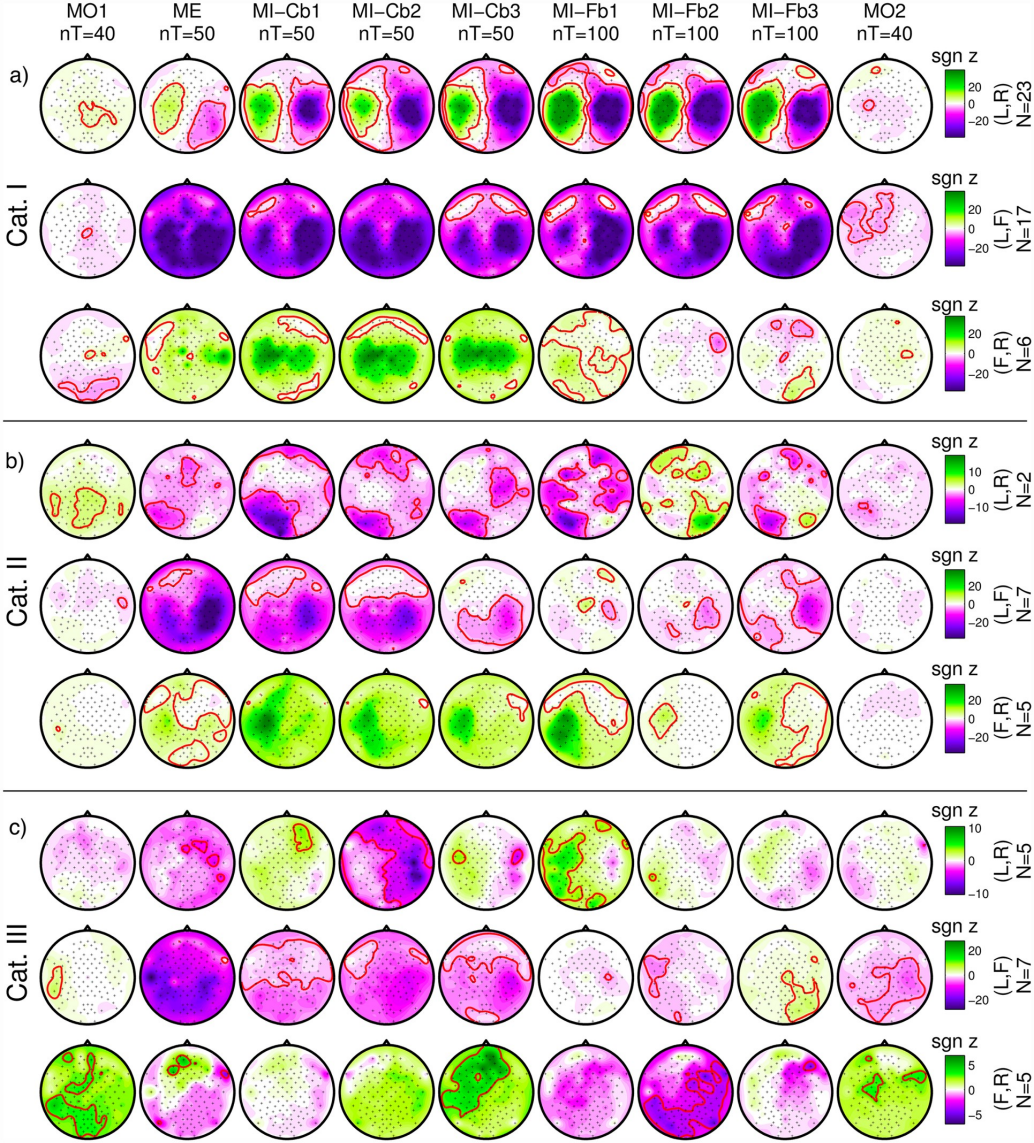
Grand-average signed *z*-scores. From left to right, the different tasks/runs, from top to bottom the class combinations and the user categories. Band power is calculated in the subject-specific frequency band and time interval. With a red line, the contour of areas with significant *p*-value (|*p*| = 0.05, which corresponds to |*z*| = 2.9957) are defined. In the middle line, red color corresponds to higher foot power whereas in the bottom line the same corresponds to green color. The scale, indicated by a colorbar on the right of each row, is adjusted in order to be the same for each row, which refers to the same number of users.

For each run and each user, the *r*^2^-values were calculated and transformed with a *z*-transformation. The signed *z*-scores are plotted as scalp maps in Fig 8.

The first three rows can be considered as exemplary, because they refer to Cat. I:

- *Cat. I, Left/Right*: the MO runs do not present a clear pattern and much lower correlation between band power and class membership. The proper motor patterns for Left/Right class combination (ERD in the contralateral hemisphere, ERS in the ipsilateral one) appear in the ME run, become much stronger in the MI-Cb runs and even stronger and more focused in the MI-Fb runs. In the last MO run a significant focused ERD in the left hemisphere can be seen.
  There might be several reasons for the weak correlation in the MO run: the MO runs have less trials than the other runs, a much higher variability of patterns among users might be present because motor observation can modulate the brain activity not only in the sensorimotor area, but also in the parietal and occipital ones and MO observation does not require active participation. Nevertheless, for the class combination *Left/Right*, a significant centro-parietal pattern can be seen with positive values, meaning a weak synchronization during the Left trials and desynchronization during Right trials.
- *Cat. I, Left/Foot*: a weak foot pattern (ERS during *Foot* observation) can be observed in the first MO run. From the ME run on, a clear ERD for the *Left* class appears, involving the ipsilateral hemisphere as well, and indicating the difficulties for many users to really desynchronize the two hemispheres. This pattern becomes stronger and more focused (much less extended in the left hemisphere) during the MI-Fb runs. A Foot pattern is not visible, probably due to two reasons: 1) Most of Cat. I users have a good ERD for the Left class and use it to obtain BCI control; 2) The Foot pattern can be either ERS or ERD so it is canceled out by averaging over subjects. The last MO run presents again a significant ERD in the left hemisphere, even more extended than for the Left/Right class combination. An ipsilateral ERD is present also in the last Foot/Right MO run. This is most probably due to the mirror effect [66].
- *Cat. I, Foot/Right*: in the first MO run, similarly to the previous row (*Left/Foot*), a significant ERS foot pattern can be seen over Cz. In fact, since *Foot* is the second class in *Left/Foot* and the first class in *Foot/Right*, the pattern found with negative values in the previous row is the same as this with positive values found for *Foot/Right*. Additionally, a significant occipito-parietal pattern is present. As for the *Left/Foot* combination, also here a significant ERD for the *Right* class involving both hemispheres appears in the ME run and becomes stronger in the MI-Cb runs whereas a *Foot* pattern is not visible by grand averaging. Differently from the previous rows, the ERD is much weaker in the first MI-Fb run and completely disappears in the subsequent runs letting visible a significant ERD in the foot area in the last MI-Fb run. Even if *z*-scores are smaller because less users were averaged for *Foot/Right* (N = 7) than for *Left/Right* (N = 23) and *Left/Foot* (N = 17), it is anyway quite clear that the right hand imagination does not provide such a stable SMR modulation as the left hand imagination so that the foot pattern develops and produces the BCI control. The last MO run presents a significant ERD in the right hemisphere, exactly in parallel to the ERD in the left hemisphere for the *Left/Foot* combination.

Also for Cat. II users, the MO patterns are different from the ME and MI patterns. It can be also observed that the 2 users with *Left/Right* combination have a parietal SMR modulation which, as already discussed, does not produce a stable BCI control. Interestingly, the same parietal pattern is shown also for the ME run, suggesting that it is not due to a wrong MI strategy, as one might suppose. Differently, users with class combinations *Left/Foot* and *Foot/Right* exhibit proper patterns, which are unfortunately not so stable during the MI-Fb runs.

Cat. III users show even more unstable patterns and no significant correlation was found in the ME runs. Nevertheless, it can be observed that from run to run for the *Left/Right* combination, proper patterns appear, and even if they are not significant, they suggest that a longer training or a better feedback (i.e. better algorithms which interpret correctly the data from the beginning and return a more stable feedback) might help these users to achieve BCI control. This solution was adopted in the *co-adaptive calibration* approach [18, 67].

#### SMR-channels

**Fig 9:**
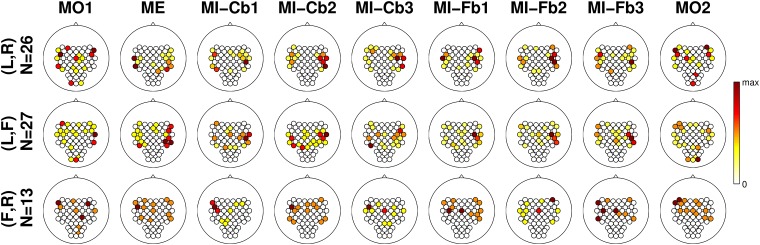
Frequency of selection of the channels as *SMR-channel*. The number of users for whom each channel was selected is color coded (white for zero, red for the maximum times of selection). From top to bottom, the three class combinations, from left to right, the nine runs.

A grand average (all 80 users) plot by class combination of the best *SMR-channel* is presented in Fig 9. The overview in Fig 9 shows which hemisphere played the main role in the BCI control. It also shows that for some users the *Foot* class was essential for the control. This was not visible from the grand average of the signed *r*^2^-values in Fig 8. For some users the *SMR-channel* fell in the ipsilateral area. The analysis of single subject scalp maps showed that this only happened to Cat. III users.

### Offline classification accuracy

**Fig 10:**
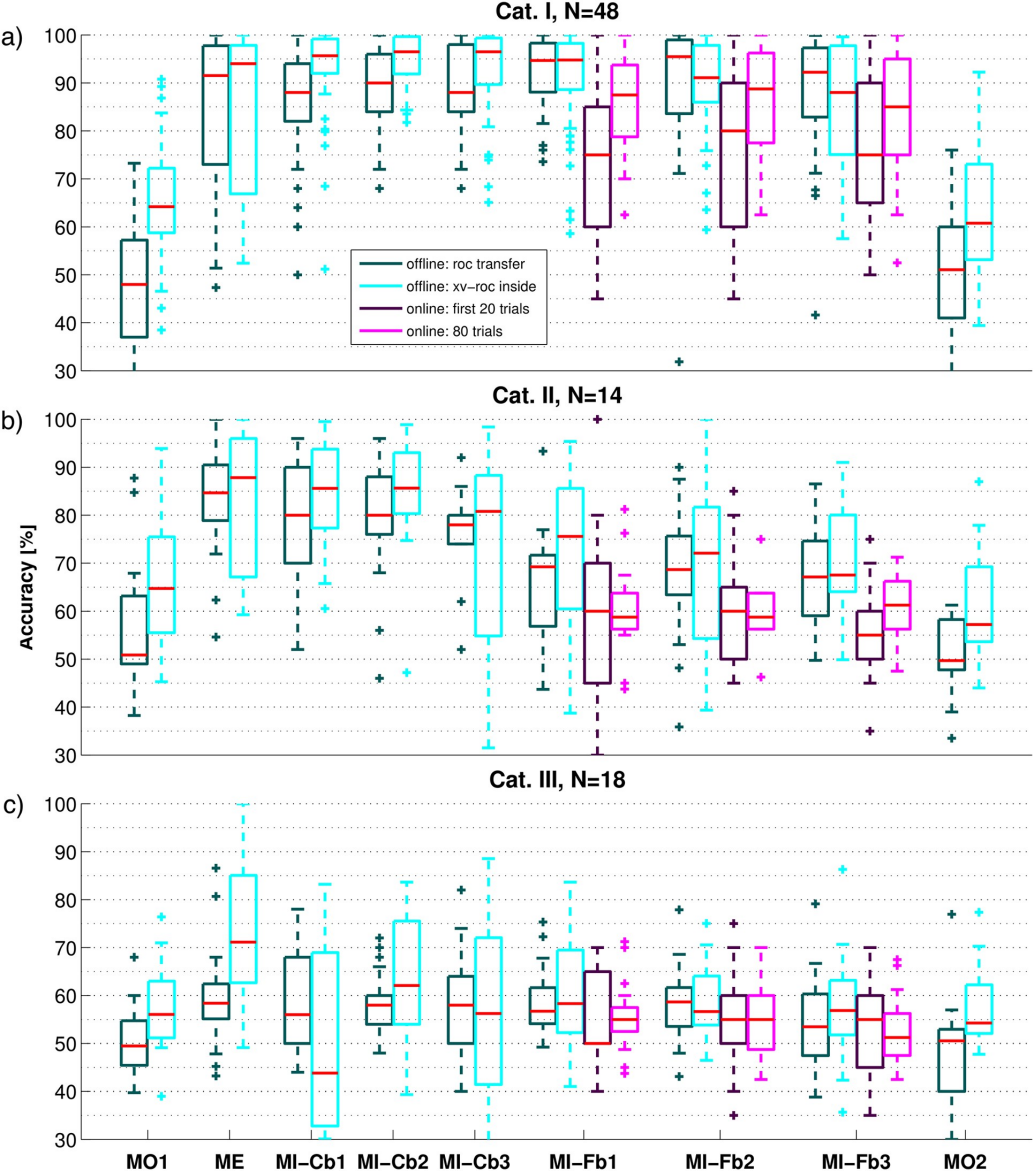
Box plots of the offline accuracy obtained for each run. The run names are indicated on the x-axis of the last row. Panels a), b) and c) correspond to Cat. I, II and III respectively. The dark cyan boxes refer to the *transfer* accuracy, obtained using subject-specific parameters, CSP filters and LDA calculated on the three MI-Cb runs during the experiment. For the offline accuracy of MI-Cb runs, CSP filters and LDA were again calculated on two MI-Cb runs and tested on the run itself. The cyan boxes refer to the *inside* accuracy, obtained selecting subject-specific band and time interval on the run itself and training CSP and LDA by LOO-CV. For the three MI-Fb runs, the online performance is also depicted: purple boxes for the first 20 trials used for bias adaptation, magenta boxes for the 80 trials relevant for the calculation of the online performance). Red line in the middle of the box indicates the median accuracy, the lower and the upper limit of the box indicate respectively the 25 and 75 percentiles of the accuracies, and the whisker length is calculated as the difference between the 75 and 25 percentiles. Accuracies outside the whiskers are considered outliers and are indicated by a cross.

Differently from a grand average analysis, which provides a global overview of the SMR patterns, the single trial classification of each data set is useful to measure the effective SNR of the data. In fact, CSP filters capture class related SMR patterns that might not be directly visible by grand average and in general by signed *r*^2^-value scalp maps. Also, up to six CSP filters might concentrate on different rhythms that developed at different moments of one run. This is especially important for the MO runs and Cat. III users: even if the SMR patterns among users are very different and thus not visible in the grand average, they might still be classifiable. Results are shown by box plots in Fig 10.

Looking at Fig 10, the following observations can be made:

- **MO vs. ME and MI runs**. The MO tasks does not produce robustly classifiable patterns, as the resulting accuracy is much lower than for ME and MI. Since the final MO run is not better classifiable than the first one, the poor classification accuracy cannot be justified by the early position of the run in the experiment. Moreover, to test whether the poor classification accuracy depends on the number of trials, the same procedures have been repeated for ME and MI runs using only the first 40 trials. Average accuracies are presented in Table 2 and again, are much lower for MO runs. This holds for all three categories.
- **ME vs. MI runs**. For Cat. III users, ME runs are much better classifiable than MI runs. This is very important because it shows that at least for part of these users, it is possible to measure classifiable SMR activity. This means that longer user training and/or improved algorithms might be helpful.
- **MI-Cb vs. MI-Fb runs**. There was no significant difference between the *inside* accuracy of MI-Cb and MI-Fb runs, both for all users considered together and for each category group separately (Wilcoxon signed rank test). Since the scalp maps in Fig 8 suggest quite stable patterns for the class combination *Left/Right*, the mean inside accuracy by class combinations was also calculated and reported in Table 3. Clearly, while for the class combination *Left/Right* the *inside* accuracy stays the same, for *Left/Foot* and for *Foot/Right* a drop happens going from the calibration to the feedback data. This is surprising because providing feedback is expected to help the user to improve his/her ERD.
- ***Transfer* vs. *inside* accuracy**. In almost all cases, the *inside* accuracy is higher than the *transfer* one. This happens especially for the MO runs, as expected by the diversity of *z*-score scalp maps in Fig 8 and frequency bands in Fig 6. For other runs which exhibit good *transfer* accuracy, but better *inside* accuracy, this means that the variability from run to run plays a more important role than the number of trials used for training the CSP filters and the LDA (higher for *transfer* than for *inside* accuracy). The contrary happens for MI-Fb2 and MI-Fb3 runs of the Cat. I, where the superiority of the *transfer* accuracy, indicates that probably the patterns deteriorate a bit with the time (because of tiredness or concentration) so that they constitute a slightly worse training set. This is also confirmed by the *z*-score scalp maps of Cat. I for class combination *Left/Foot* and much more for *Foot/Right*. Nevertheless, for Cat. II and Cat. III, the variance of *inside* accuracy is always much larger than for the *transfer* one, indicating that overfitting occurs in the selection of subject-specific parameters and CSP filters. The *transfer* accuracy is much higher also for MI-Cb1 for Cat. III, indicating a strong overfit when 50 trials are used as training set. For the other runs of Cat. III, *transfer* and *inside* accuracies do not differ so much, indicating that even algorithms trained on the trials of the same run, cannot improve the performance of these users.
- **Feedback Runs, online vs. offline**. The online accuracy of the MI-Fb runs is always worse than the offline one. This points out an important timing problem, since the offline accuracy is calculated using the subject-specific time interval, while the online classification is determined at the end of the trial. Moreover, it can be noticed that for Cat. I and Cat. II, the offline accuracy, especially the *inside* one, decreases from run to run because the actual SMR patterns deteriorate. Differently, for Cat. I the *online* accuracy increases from MI-Fb1 to MI-Fb2, showing a clear user’s learning effect and adaptation to the trial timing and then decreases in MI-Fb3 probably because of tiredness (*p* = 0.028 by Wilcoxon signed rank test for the equality of median between MI-Fb1 and MI-Fb3). For Cat II, the online accuracy increases in the last run (MI-Fb3) showing that these users need more time than Cat. I participants to learn the task. Learning is not possible for Cat. III users, whose accuracy does not show any improvement but rather a drop in the last run, MI-Fb3.
- **Bias Adaptation**. The bias adaptation after the first 20 trials is effective just for Cat. I users. The reason might be that, given the high non-stationarity or noise of the data in Cat. II and Cat. III users, the bias adaptation is not successful and maybe even worsens the classification.

**Table 2:** *Inside* performance of each run averaged by category.

Cat.	MO	ME	MI-Cb.	MI-Fb.	MO
I	64.20	89.89	95.61	85.89	60.93
II	65.24	76.39	87.42	65.06	58.73
III	57.03	67.80	47.22	53.92	54.28
All	63.50	79.78	87.76	74.72	59.75

*Inside* performance calculated by LOO-CV on the first 40 trials of each run and averaged by user category. The last row shows the average across all 80 users.

**Table 3:** *Inside* accuracy of calibration and feedback data by category.

	Calibration Inside AUC [%]				Feedback Inside AUC [%]			
	Cat. I	Cat. II	Cat. III	Mean±Std	Cat. I	Cat. II	Cat. III	Mean±Std
LR	93.66	91.46	64.03	88.58 ± 13.55	94.85	91.75	63.75	89.46 ± 14.10
LF	94.18	84.46	64.47	85.19 ± 14.69	89.85	81.52	64.85	82.25 ± 15.11
FR	93.33	83.50	60.46	79.99 ± 16.98	89.08	75.79	60.99	76.15 ± 14.48
All	93.83	85.12	63.23	85.42 ± 14.90	92.15	80.93	63.47	83.73 ± 15.27

*Inside* accuracy (calculated by LOO-CV on each run) of calibration and feedback data averaged by user category and class combination. The mean values across runs of the AUC is reported. The last row shows the average across all class combinations.

The classification accuracy re-calculated by 8-fold CV (as done during the experiment) and the online feedback performances averaged by runs and categories are additionally reported in Table 4, since they are usually more important for comparison with new developed methods which make use of calibration and feedback data.

**Table 4:** Offline calibration accuracy and feedback performance.

	Calibration [%] (offline)				Feedback [%] (online)			
Cat.	Run 1	Run 2	Run 3	Mean±Std	Run 1	Run 2	Run 3	Mean±Std
I	90.77	91.34	90.85	90.99 ± 6.16	86.54	86.62	82.85	85.60 ± 9.94
II	81.79	81.78	80.08	81.22 ± 8.24	60.36	61.15	60.42	61.79 ± 8.60
III	64.91	64.97	63.49	64.46 ± 6.64	55.19	55.87	53.27	54.40 ± 4.97
All	79.16	79.36	78.14	83.31 ± 12.68	74.97	75.21	71.75	74.42 ± 16.48

Offline calibration accuracy (re-calculated by 8-fold CV as during the experiment) and online feedback performance obtained during the experiment averaged by runs and user categories. The last row shows the average across all 80 users.

### SMR-predictor analysis

#### SMR-predictor grand average analysis

The SMR-predictor was extracted using the condition *relax with eyes open* from the artifact recordings.

**Fig 11:**
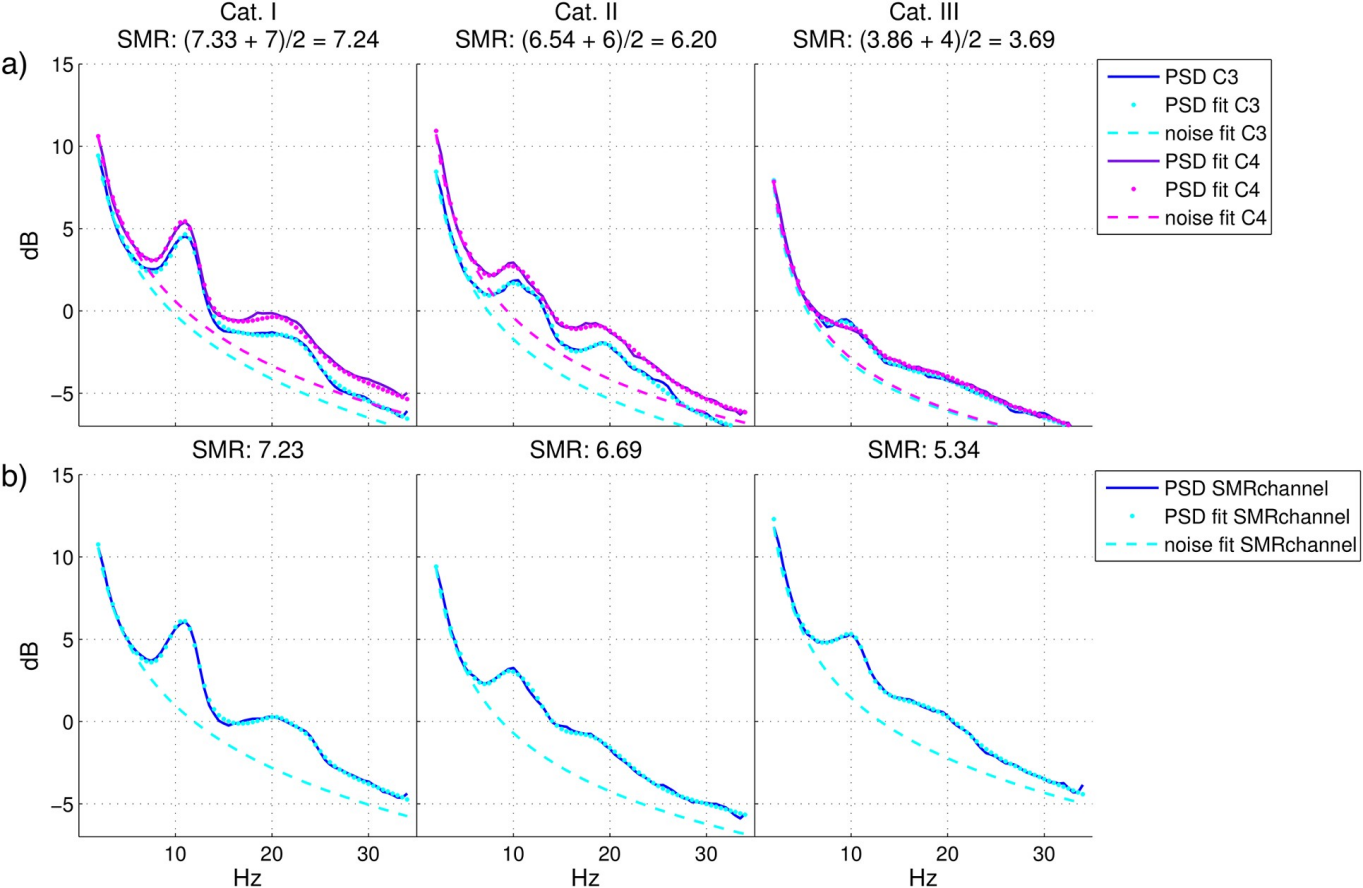
Spectrum at rest and PSD fit. Spectrum at rest (PSD, solid line), model of the PSD (dot line) and model of the noise (dashed line) averaged across users belonging to the same category. From left to right, Cat. I, Cat. II and Cat. III. Panel a): Laplacian derivation of C3 (PSD in blue, PSD fit and noise fit in cyan) and C4 (PSD in purple, PSD fit and noise fit in magenta). In the title, the mean *SMR-strength* values (the first one is for C3, the second one is for C4) and the SMR-predictor value are reported, calculated separately for each user and channel and then averaged. Panel b): Laplacian derivation of the *SMR-channel*, which is subject-specific.

In Fig 11, the grand average per categories of the PSD, the $\hat{PSD}$, the noise fit and the SMR-predictor is shown. It can be observed that, as expected, the PSD of C3 and C4 for Cat. I is exemplary with two clear peaks in the μ (around 12 Hz) and *β* band. For Cat. II, both peaks happen 2-3 Hz earlier (around 10 Hz) and are much smaller while Cat. III plot exhibits an almost flat spectrum with a very small peak also around 10 Hz. More interestingly, both PSD and noise are consistently (but not significantly) higher for C4 in comparison to C3, but the contrary happens for the *SMR-strength*, i.e. the maximum distance between the $\hat{PSD}$ and the noise fit, which is higher in C3. In order to confirm these observations, statistical tests were conducted to investigate the correlation of the feedback performance with 12 different variables. Those variables were calculated at the *f*_*max*_ which maximizes the difference between $\hat{PSD}$ and the noise fit (i.e. *f*_*max*_ is different for each user and channel): $\hat{PSD}$, noise, frequency *f*_*max*_ itself and *SMR-strength* in C3 and C4 (8 variables) and mean of $\hat{PSD}$, mean of noise, difference of *f*_*max*_ and mean of the *SMR-strengths* over the two channels (the last one is thus the SMR-predictor). Pearson or Spearman correlations were calculated depending on the result of the Lilliefors test for normal distribution [68]. All correlations resulted significant (*p* < 0.05) except for the noise and the *f*_*max*_ in C4. While a feature ranking is not the aim of this analysis, it is surprising to note that: 1) C3 seems to be more relevant than C4. In fact, differently from C4, the noise and the *f*_*max*_ in C3 are significantly correlated with the BCI feedback performance (*p* < 0.001 resp. *p* < 0.05). Moreover, the three variables with normal distribution ($\hat{PSD}$, noise fit and *SMR-strength*) for C3 and C4 were transformed to binary to be used as input for a two-way analysis of variance by ANOVA, one test for each pair of variables. Results showed no interaction between values in C3 and C4, but always higher significant effect for C3. 2) The mean of $\hat{PSD}$ and the SMR-predictor over C3 and C4, had higher correlation than C3 and C4 alone, meaning that both channels had separated (no interaction) significant effects on the performance. 3) The difference between the *f*_*max*_ in C3 and C4 was also high significantly correlated with the feedback performance (*p* < 0.001) and as already stated before, even the *f*_*max*_ in C3 alone (*p* < 0.01). Moreover, these correlations were negative (also for C4 *p* = 0.09), in line with already presented results, where a feedback based on lower frequencies is more robust than a feedback based on frequencies in the *β* or *γ* band.

On panel b) of Fig 11, the PSD, the PSD model, the noise fit and the *SMR-strength* are calculated using the subject-specific *SMR-channel*. The *SMR-strength* for this channel is higher for Cat. II and especially for Cat. III users, whose spectra clearly exhibits a peak around 10 Hz. This indicates that at least some users of Cat. III have SMR activity but not in the expected positions and this is visible already in the EEG at rest [19, 20].

#### Effective Cat. III users by SMR-predictor

**Fig 12:**
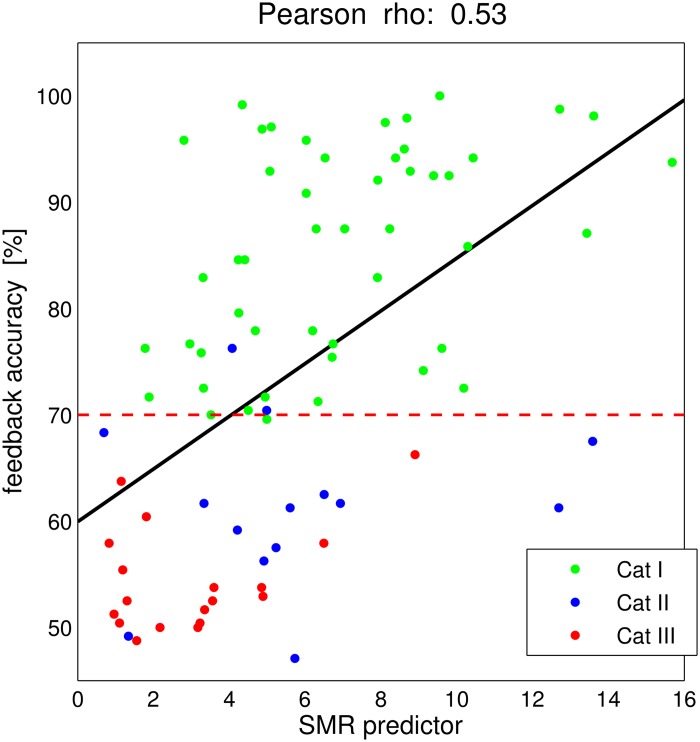
SMR-predictor results. The SMR-predictor is visualized as in [17] with the addition of the user categories. One dot per subject. Linear regression between SMR-predictor values and average feedback accuracy results in the black line. Pearson correlation coefficient *r* = 0.53. The criterion level of feedback accuracy of 70% is marked by a red dashed line.

In Fig 12, the BCI feedback performance (average across the three MI-Fb runs) versus the proposed SMR-predictor is depicted. The SMR-predictor explained as much as *r*^2^ = 28% of the variance in the feedback accuracy in our sample of 80 participants. The dashed red line indicates the performance threshold of 70%, under which the users are in Cat. II and Cat. III. Following these results, one can expect users with a SMR-predictor higher than 3 (or even 3, considering the green dots with lowest SMR-predictor values) to be able to reach BCI control. In Fig 12, there are several users with feedback performance below 70% and relatively high SMR-predictor values. It can be hypothesized that those users have the potentiality to obtain a better BCI control under new training strategies or new algorithms for EEG online processing and classification. In fact, only 9 of the Cat. III users have an SMR-predictor lower than 3. This corresponds to 89% of the total number of users.

### Psychological predictors of BCI performance

The results of the psychological tests were analyzed in collaboration with the Tübingen group and published in [33]. In this analysis subjects were not divided in categories but considered all together. Statistical analyses allowed us to select those variables significantly correlated with the feedback performance (called BCI performance). Additionally, a logistic regression model was constructed with the most significantly correlated variables to estimate how much the variance of the BCI performance they could explain. Due to a significant drop in the performance in the third run and the earlier interruption of the experiments by several participants, the BCI performance used for the statistical analysis was the mean value of the performance in MI-Fb runs 1 and 2. The results showed that different imagery strategies did not lead to any significant differences in BCI performance but the ability to concentrate on the task was significantly correlated with the BCI performance.

#### Runwise test

The runwise tests variables cannot be used as performance predictors, since the corresponding questions were asked after each run. Still, their result helps to explain the performance drops. Among the asked variables *tiredness*, *motor imagination strength*, *motivation*, *anger*, and *uneasiness*, all except for *anger* were highly significant correlated (Spearman correlation) with the feedback performance: tiredness with *r* = -0.18, *p* < 0.05, MI strength with *r* = 0.44 and *p* < 10^−9^, motivation with *r* = 0.29 and *p* < 0.001, and uneasiness with *r* = -0.25 and *p* < 0.01. Bonferroni correction was applied by multiplying the *p*-values by 5.

## Discussion

### General comments

Despite the great progress over the last years many BCI studies focused on the improvement of the signal processing algorithms and only recently the inter-subject variability and the individual characteristics that might correlate with the BCI performance have been taken into account (see e.g [69]). Finding such correlates is useful in order to establish from the beginning a subject-adapted training strategy, from the algorithmic side and/or the paradigmatic one, and to avoid long frustrating BCI training sessions for those users for whom the SMR-based BCI might be unsuitable.

Large scale studies are needed to take into account the wide variation of users and to obtain robust statistics. Some previous works [14, 41, 42] conducted in noisy environments (such as exhibitions) were useful to assess how many people are in general able to use BCI, but short questionnaires and no EEG data analysis were conducted for a deeper analysis.

Here, a detailed screening study conducted in collaboration with the University of Tübingen was described. A population of 80 BCI naive participants underwent a BCI session with MO, ME and MI runs accompanied by psychological tests before, during and after the experiment. Some days before the BCI session, the participants also took part in a psychological test-battery (2-3 h). In Table 5 we present a summary of the results described in previous sections.

**Table 5:** Summary of results according to categories.

Result	Cat. I	Cat. II	Cat. III
Classif. accuracy	Cb and Fb ≥ 70%	Cb ≥ 70% and Fb < 70%	Cb < 70%
SMR modulation	At least for one class and stable	Exists but unstable	Does not exist
ME and MI	MI easier to classify (less ipsilateral ERD)	MI and ME very similar	ME is classifiable but MI not
Reactive band	Stable over time L/R mostly *μ* band *β* only for F	Changes from Cb to Fb *μ* or *β* bands	Changes over time
SMR channel	Located as expected	Sometimes parietal (unstable)	Located in unexpected positions (ipsilateral hemisphere)
Transfer vs. Inside	Inside better	Large variance inside (overfit)	Large variance inside (overfit)
Offline vs. online	Adjust timing in 1 run	Two runs to adjust timing	No improvement over time
Bias adaptation	Effective	Ineffective	ineffective
SMR-strength	Stronger in C3 than C4 and at SMR-channel	Stronger at SMR-channel than at C3 and C4	Much stronger at SMR-channel than at C3 and C4
SMR-predictor	Visible peaks at 12 Hz	Visible peaks at 10 Hz	Small peaks or none at 10 Hz andpredictor below 3

### BCI user categories

For the first time, a detailed categorization of BCI users was introduced depending on their calibration and feedback performances. The majority of users (60%) belonged to Cat. I, with calibration and feedback performance both higher than the criterion level of 70%. Cat. II was assigned to those users (17%) who had a good calibration performance but poor (below 70%) feedback performance. These users developed in general an inefficient MI strategy or had problems in the step from calibration to feedback. The rest of the participants (22.5%) exhibited non-classifiable data already in the calibration session so that also the feedback performance was lower than 70% or impossible to assess.

Based on their SMR activity, also a systematic list of the reasons which lead to a performance drop and thus hints about possible improvements for the BCI experimental design are given. The categorization of a user is very useful 1) to adopt a more successful training strategy, 2) to select users for further analyses as well as for testing new BCI paradigms or algorithms, and 3) for a better comparison between different studies. In fact, it is usual to report the mean performance across all users without taking into account that, sometimes just good BCI performers took part in the study.

The single-trial classifications under different parameter settings and training set sizes revealed that overfitting occurs for Cat. II and Cat. III users, whereas this seems not to be a problem for Cat. I ones. More importantly, Cat. II and Cat. III users often develop more efficient SMR patterns during feedback runs, as *z*–scores scalp maps show. These changes, as well as changes in the reactive frequency band and timing problems (short or late ERD) cannot be solely solved by improving the algorithms trained on the MI-Cb data to classify the MI-Fb ones. In fact, several attempts have been done to optimize subject-specific feature extraction [70, 71], to render CSP filters invariant to non task relevant EEG changes [72–76] or by regularizing it against overfitting [77, 78]. All these approaches use the calibration data as training set and can improve in general the classification performance, but cannot always predict the patterns that appear in the feedback sessions. As observed in the Results section, even the bias adaptation of the LDA classifier after 20 trials of feedback, as described in [79], did not succeed for users of Cat. II and Cat. III. Online adaptation of the classification algorithms on the contrary offers a solution to this problem. Newly developed techniques for unsupervised adaptation of the LDA classifier using the density distribution of the feedback data [80] or [81], although not suitable to capture changes in the reactive frequency band and SMR patterns, adapt efficiently to changes in the background activity, given that the activation patterns stay the same. Offline and online experiments using covariate shift adaptation showed improvement in classification accuracy in presence of non-stationarities [80, 82]. More recently, in [18, 19, 67, 83] it has been shown that the development of proper SMR patterns is facilitated by better feedback and is also frequent in Cat. III users [19].

The grand average of Cat. III users did not exhibit significant correlations between band power and class membership even during the ME runs, indicating that the difficulty encountered by the majority of these users to achieve BCI control is related to intrinsic properties of their EEG activity. Nevertheless, from run to run for the *Left/Right* combination, expected patterns appear, and even if they are not significant, they suggest that a long training or a better feedback (i.e. better algorithms which interpret correctly the data from the beginning and return a more stable feedback) might help these users to achieve BCI control. This is confirmed by the single trial offline classification of the ME run, which delivered a higher accuracy (median across subjects above 70%) than for the MI runs.

### Limb prevalence

The majority of Cat. I users employed the combination *Left/Right* and showed ERD/ERS for both hands. The *Foot* class came into play when the desynchronization between left and right hemispheres resulted difficult. This phenomenon is much larger and stronger for the *Left/Foot* combination, and it is also visible in the feedback runs. This, together with the stronger *SMR-strength* observed on C3 in comparison to C4, leads to the hypothesis that these users face some difficulty to disengage the left hemisphere, resulting in a larger ERD and better calibration performance for *Left/Foot* than for *Foot/Right*. This would explain why the combination *Left/Foot* is more often chosen than *Foot/Right* (34 vs. 16 users), so that class *Left* was more frequently selected (64 users) than *Right* and *Foot*. This is in line with [84, 85] and [86], where a dominance of the left hemisphere is observed by larger ERDs around C3.

### Reactive frequencies

The analysis of the subject-specific frequency bands revealed that participants who obtained the BCI control by *Left* and/or *Right* hand motor imagery mostly did it by modulating the *μ* band, while the *β* band has been selected when the control was achieved by imagination of the *Foot*. The performance was significantly higher for users who employed the *μ* SMR. It is difficult to assess whether this is due to a higher stability of the *μ* SMR in comparison to the *β* SMR, or to the fact that hand and foot pattern were often not captured in the same time interval of the trial when they appeared in two different bands. In fact, the SMR activity, the reactive frequency bands and the classification accuracy resulted more stable across runs for the *Left/Right* class combination.

### Motor observation

The MO runs did not exhibit classifiable patterns, even for Cat. I users. This is in contrast with previous studies [87–90] which led to the “mirror neuron theory” hypothesized in [91]. This theory described an observation/execution matching system, in which the activity of mirror neurons reacting to motor observation, modulates the premotor neurons that are then reflected by the modulation of *μ* rhythms as well. It should be noted that in [90] and in [89], just differences between the SMR in the EEG at rest and in the EEG during motor observation were observed, but no data classification was carried out. In [86], the participants performed motor imagination with a realistic feedback, thus motor imagery and observation occurred at the same time. This is essentially different from the task presented here, where the participants carefully observed videos and imagined that the represented limbs were their own. The classification results between motor imagery with realistic or abstract feedback did not differ. In [45] the users performed motor imagery during motor observation. Finally, in [51] better discrimination of MO data was reported in comparison to MI. Nevertheless, the depicted SMR activity was mainly parietal, which is not in line with [90] and [89]. The better discrimination of the MO data reported in [51] might be due to the difference in the feature selection algorithms. In particular, they classified a combination of features in several frequency bands, whereas in this manuscript we applied CSP which is particularly successful for ERD features.

### Relax recordings

The relax recording revealed to be very important to monitor the basic potentiality of a user to use BCI. The SMR-predictor [17] resulted a very useful tool to categorize the users and estimate from the beginning of the experiment how good they might perform motor imagery. Moreover, since for Cat. II and Cat. III users the subject-specific *SMR-channel* had a higher *SMR-strength* than C3 and C4 already in the relax recording, it can be hypothesized that the relax recording itself might be used to select subject-specific areas with higher SMR potentiality and use them as information to optimize the BCI system. Similarly, the relax recording and the PSD model can be utilized to estimate the subject-specific reactive frequency band already before starting the BCI session. Interestingly, the peak in the relax spectrum for Cat. I users was around 12 Hz, while the peak in the relax spectrum of Cat II and Cat III users was around 10 Hz. In [51], it was observed that the low *μ* component was responsible for a general but not class-specific ERD, whereas the high *μ* allowed the classification between left and right. Again, a dominance of the left hemisphere is confirmed by a higher peak, stronger *SMR-strength* and a more significant correlation of parameters (reactive frequency, noise level and peak amplitude) with the feedback performance in C3 than in C4.

## Conclusion

This paper presents a detailed analysis of a large dataset of 80 novice SMR-BCI users who performed an experiment with standard machine learning based system, i.e. with calibration and feedback runs. The causes identified for poor BCI control explain this problem for a significant portion of participants (40%). This deep analysis allowed us to define three different Categories of participants. The absence of an SMR peak in *μ* and/or *β* bands seems to be a signal characteristic intrinsic to the user. Indeed, as shown in [17] a more or less pronounced peak in the spectrum of the EEG data at rest can predict the BCI performance with certain reliability. The question whether further training would increase the peaks at rest or improve the SMR-modulation during an experimental BCI session may be worth further investigation. Nevertheless, there is some evidence pointing to the possibility that Cat. II and Cat. III users can learn to modulate motor imagery rhythms and achieve BCI control when the system used is co-adaptive in the feature and classifier spaces [18, 19, 67]. In particular, about 9 users have an SMR-predictor lower than 3. This means that in principle, algorithms should be able to find rhythmic features for the rest 89% of SMR-BCI users. The analysis of individual CC-FA within a DTI study in [92] also revealed specific differences between good and poor SMR-BCI performers. However, this neurophysiological property can just predict 28% of the variance in the BCI performance across 80 users and the anatomical structure predicts just 34% of the variance across 20 pre-selected users. These data indicate that the rest of the variance is imputable to other reasons such as the algorithms or the paradigm [19, 32]. Moreover, clear evidences of neural plasticity in humans induced by BCI feedback have been recently demonstrated [93] also in clinical applications [94], so that it is reasonable to assume that a better BCI feedback would lead also users with small SMR amplitudes to enlarge the motor areas involved in the motor task, resulting in a higher SMR peak at rest and an easier SMR modulation. Here we want to remark, that we studied the particularities of EEG signals from many BCI users in order to understand how to improve the efficiency of BCI systems. In the past, users for whom BCI control was not successful (even with the state-of-the-art algorithms) were referred to as “BCI illiterates” by some authors. However, this term lays the responsibility of poor BCI control on the user rather than on the system. As explained in [95], expressions like “BCI illiteracy” or “BCI illiterates” should be avoided. Furthermore, in our paper [18] we already employed the phrase “BCI efficiency” to emphasize that it is the system which needs to be improved and better guide the user to achieve sufficient performance. Indeed, we believe it is responsibility of the scientists to refine BCI methodologies until all possible users are able to gain control.

In the future we would also like to investigate the effect of re-referencing in the results presented in this paper. In this work we used Laplacian derivations, which is not consider a re-referencing procedure because it does not change the usual EEG voltages. However, recent papers have shown that the method chosen to re-reference EEG data can have a great impact in the results obtained in EEG analyses, [50, 53–56]. Thus, it should be investigated to which extent re-referencing can have an impact in the extraction and analysis of SMR modulations, specially in those cases where SMR modulations are hardly observable.

Concluding, this work represents a detailed attempt to give an overview on the BCI inefficiency problem for SMR-BCIs, locating the shortfalls from the neurophysiological and algorithmic point of view in the experimental standard approach.
